# A genetic approach for analyzing the co-operative function of the tRNA mimicry complex, eRF1/eRF3, in translation termination on the ribosome

**DOI:** 10.1093/nar/gku493

**Published:** 2014-06-09

**Authors:** Miki Wada, Koichi Ito

**Affiliations:** 1 Technical office, The Institute of Medical Science, The University of Tokyo, Minato-ku, Tokyo 108-8639, Japan; 2 Department of Medical Genome Sciences, Graduate School of Frontier Sciences, The University of Tokyo, Kashiwa-city, Chiba, 277-8562, Japan

## Abstract

During termination of translation in eukaryotes, a GTP-binding protein, eRF3, functions within a complex with the tRNA-mimicking protein, eRF1, to decode stop codons. It remains unclear how the tRNA-mimicking protein co-operates with the GTPase and with the functional sites on the ribosome. In order to elucidate the molecular characteristics of tRNA-mimicking proteins involved in stop codon decoding, we have devised a heterologous genetic system in *Saccharomyces cerevisiae*. We found that eRF3 from *Pneumocystis carinii* (Pc-eRF3) did not complement depletion of *S. cerevisiae* eRF3. The strength of Pc-eRF3 binding to Sc-eRF1 depends on the GTP-binding domain, suggesting that defects of the GTPase switch in the heterologous complex causes the observed lethality. We isolated mutants of Pc-eRF3 and Sc-eRF1 that restore cell growth in the presence of Pc-eRF3 as the sole source of eRF3. Mapping of these mutations onto the latest 3D-complex structure revealed that they were located in the binding-interface region between eRF1 and eRF3, as well as in the ribosomal functional sites. Intriguingly, a novel functional site was revealed adjacent to the decoding site of eRF1, on the tip domain that mimics the tRNA anticodon loop. This novel domain likely participates in codon recognition, coupled with the GTPase function.

## INTRODUCTION

Termination of translation in eukaryotes is catalyzed by two classes of polypeptide release factors, eRF1 (class I) and eRF3 (class II) (1–3). eRF1 recognizes stop codons directly and activates mature polypeptide release by peptidyl-tRNA hydrolysis (4–6). eRF3 is a G-protein that is related to translation elongation factors (EFs), and stimulates polypeptide release by eRF1 (7,8). eRF1 and eRF3 heterodimerize. This binding between eRF1 and eRF3 has been studied extensively (1,2,9,10), and the C-terminal domains of both eRF1 and eRF3 were shown to be sufficient for this interaction. In mutational analyses, the binding between eRF1 and eRF3 correlated with their co-operative functionality (11). Biochemical studies revealed that eRF1•eRF3•GTP complex formation is strongly enhanced in the presence of Mg^2+^ (12,13) and that the GTPase activity of eRF3 is stimulated by eRF1 and the ribosome (7).

X-ray crystal structure analysis of eRF1 has revealed that it is composed of three domains, namely, N, M and C (14), where domain N comprises residues 1–138, domain M comprises residues 139–271 and domain C comprises 272–431 in *Saccharomyces cerevisiae*. Domain N is primarily responsible for codon recognition, and the amino acid motifs TASNIKS and Y-C-F within domain N have been reported to be important for codon recognition (5,15). Unlike class I release factors in prokaryotes, eRF1 is able to decipher all stop codons (UAA, UAG and UGA), although the precise recognition mechanisms remain to be clarified. Domain M contains the universally conserved GGQ motif that corresponds to the CCA-end of tRNA (4,5,16) and plays a role in peptide release from the peptidyl-tRNA by activating hydrolysis at the peptidyl transferase center of the ribosome (14).

eRF3 is composed of two distinct regions: the N-terminal domain (residues 1–253 in *S. cerevisiae*) and the C-terminal region (17,18). The N-terminal domain *per se* is less conserved among species and is dispensable for translation termination as well as for viability of yeast cells, and is thought to modify the catalytic action of the C-terminal region (19,20). The C-terminal region of eRF3 (eRF3c) is responsible for translation termination and is highly homologous to EF1α (EF-Tu in prokaryotes) (1,9). The X-ray crystal structure of eRF3c revealed that it is composed of three domains, namely, 1, 2 and 3, as is EF1α (11). Domain 1 comprises residues 254–488, domain 2 comprises residues 489–576 and domain 3 comprises residues 577–685 in *S. cerevisiae*, with domain 1 corresponding to the G-domain that contains conserved G-protein motifs (21).

Despite considerable efforts to obtain the X-ray crystal structure of the eRF1 and eRF3 complex, only structures of the complex lacking the G-domain (i.e. domain 1) have been solved (22). This has confirmed that complex formation between eRF1 and eRF3 is largely dependent on binding of the proteins via their C-terminal domains.

Recently, the archaeal EF1α (aEF1α) from *Aeropyrum pernix* has been shown to play versatile roles in multiple translational steps, such as elongation and termination, and also in mRNA quality control (23), and can form a complex with tRNA, aRF1 (archaeal class I RF) and Pelota (Dom34 in *S. cerevisiae*) (24,25). Furthermore, the archaeal aEF1α complexes exhibited competitive binding to tRNA, aRF1 and aPelota. Thus, the archaeal translation termination complex aRF1/aEF1α is comparable to the eukaryotic eRF1/eRF3. These finding strongly suggested that the carrier GTPase protein aEF1α can bind to both tRNA and the binding protein factors, and can function on the ribosome in a similar way.

On the other hand, X-ray crystal structures of archaeal aEF1α•aPelota•GTP (25) and aEF1α•aRF1•GTP (26) complexes have been solved, and both of these complex structures showed striking structural similarities to the EF-Tu/tRNA complexes. These structures revealed two major interaction sites between archaeal class I and class II release factors, named site 1 and site 2; these sites are probably also relevant to eukaryotes. Site 1 is a classical binding site between the two C-terminal regions, and site 2 is the G-domain interaction site. The concept that release factors share catalytic sites on the ribosome with tRNAs and/or EFs during codon decoding was previously termed the ‘RF-tRNA mimicry hypothesis’ (27). In eukaryotes, a common mechanical basis underlies all these decoding processes, including an as-yet-unknown process for mRNA surveillance.

The X-ray crystal structure of the 70S ribosome•EF-Tu•tRNA complex (28) and cryo-EM structures of the 80S ribosome•Dom34•Hbs1•GDPNP complex (29) and the 80S ribosome•eRF1•eRF3•GDPNP complex (30) have recently been reported. These structural studies have provided much information about the step-by-step functions of the translation factors on the ribosome. Despite the striking tRNA mimicry, however, the crucial molecular mechanisms as well as the functional domains of eRF1/aRF1 in the ribosome remain poorly understood. Uncovering the molecular mechanisms from a functional point of view will require biochemical and genetic analyses.

We have previously reported cDNA cloning of eRF genes from *Pneumocystis carinii* (31). *Pneumocystis* is an opportunistic pathogen that causes severe pneumonia in immunocompromised hosts (32,33). *Pneumocystis* is classified as a unique fungus; phylogenetically, its closest well-known relative is *Schizosaccharomyces pombe* (34–36). *P. carinii* is one of the best-studied *Pneumocystis* species, and preferentially infects rat. However, little is known about protein synthesis in *P. carinii*. It is known that *P. carinii* harbors a single copy of the 5S rRNA gene on its genome, suggesting a diverged translational system (37), which may reflect the unique niche of the organism in the natural environment.

In this study, we found that eRF3 derived from *P. carinii* (Pc-eRF3) cannot replace endogenous eRF3 in *S. cerevisiae*, despite earlier reports that mammalian eRF3 orthologs can replace that of *S. cerevisiae* (11,38). We report genetic mapping and analyses of the critical sites that contribute to functional complementation of Pc-eRF3 in yeast, in order to elucidate the functional interplay among eRFs and the ribosome.

## MATERIALS AND METHODS

### Strains and media

*S. cerevisiae* strains used in this study are listed in Supplementary Table S1. The tet-OFF eRF3 (*SUP35*) strain was constructed as per the method described previously (24), using hphMX4 as selection marker (39). The double tet-OFF eRF1/eRF3 strain was constructed by replacing the *SUP45* (eRF1) gene promoter of the tet-OFF eRF3 strain with the tet-OFF promoter along with the kanMX4 selection marker. Manipulation of yeast and plasmid DNA was performed according to standard procedures (40,41). Media for yeast were YPD or synthetic complete media, prepared with the appropriate dropout mix (ForMedium™; Hunstanton, UK), and for plates, 2% agar was added. Manipulation of *Escherichia coli* was performed as described previously (41).

### Plasmid construction

Plasmids and primers used in this study are listed in Supplementary Tables S2 and S3, respectively. Full-length Pc-eRF3, Pc-eRF3c and Δ1 DNA fragments were amplified by polymerase chain reaction (PCR) from a Pc-eRF3 cDNA clone (DDBJ AB052894) (31) using primer pairs P363/P318, P362/P318 and P555/P318, respectively, and were introduced into the indicated vectors using BamHI/SalI restriction enzyme sites. The K349A Pc-eRF3c mutation was introduced by site-directed mutagenesis, using DNA primers P549 and P550. The Sc-eRF3c DNA fragment was amplified by PCR from the *S. cerevisiae* genomic DNA with primers P291/P292 and were cleaved with BamHI/XhoI sites before being cloned into BamHI/SalI-cleaved vectors. The Sc-eRF1 DNA fragment was amplified by PCR from the *S. cerevisiae* genomic DNA with primers P289/P306 and were cloned into the vectors via BamHI/SalI sites. The internal BamHI site of Sc-eRF1 gene was removed by a silent mutation designed in the P306 primer. For FLAG-tag fusion protein expression, FLAG-tag encoding DNA fragment was generated by annealing and amplifying the P420/P421 oligonucleotides and inserted this into vectors p416GPD and p416CYC, to fuse in-frame with the open reading frames (ORFs) at the N-terminal, via XbaI/BamHI sites.

### Complementation analysis

The tet-OFF eRF3 strain was transformed with eRF3 expression plasmid vectors based on p416GPD, and the growth of transformants was monitored on SC–Ura plates containing 7.5 μg/ml doxycycline. The double tet-OFF eRF1/eRF3 strain was co-transformed with the wild type or the mutant vector plasmids of Sc-eRF1 in p414GPD, as well as with eRF3s in p416GPD, and the growth of transformants was monitored on SC–Ura–Trp plates containing 7.5 μg/ml doxycycline.

### Western blot analysis

Protein expression levels of Pc-eRF3 variants in the tet-OFF eRF3 strain was monitored by western blot analysis using an anti-FLAG antibody, Monoclonal ANTI-FLAG M2 (F3165) (Sigma-Aldrich, St. Louis, MO, USA) against the cell extracts from p416GPD-FLAG-Pc-eRF3s transformants. Expression levels of eRF3 variants, fused to the GAL4 binding domain via the yeast two-hybrid analysis vector, pGBT9, were monitored using an anti-GAL4BD antibody, GAL4 antibody (DBD) (sc-577) (Santa Cruz Biotechnology, Dallas, TX, USA) against the cell lysates from AH109 transformants, as follows. Transformants were grown in the appropriate liquid media to mid-log phase. The yeast cells were collected and precipitated with 10% trichloroacetic acid, suspended in sodium dodecyl sulphate (SDS) sample buffer, neutralized with 5 N NaOH solution, and vigorously mixed with glass beads on the FastPrep 24 instrument (MP Biomedicals, Santa Ana, CA, USA). Proteins in the cell extracts were separated by SDS-polyacrylamide gel electrophoresis (SDS-PAGE), transferred to polyvinylidene difluoride (PVDF) membranes and detected using the appropriate antibodies and an ECL Western Blotting Detection System (GE Healthcare, Little Chalfont, UK).

### Yeast two-hybrid analysis

Wild-type or mutant eRF1 and eRF3 DNA fragments were cloned in-frame into the activation domain vector pGAD424 and the DNA-binding domain vector pGBT9, respectively, for expression as fusion proteins. The AH109 yeast two-hybrid reporter strain was transformed with both plasmids and transformants were then examined for growth on SC–Leu–Trp–His plates (Clontech Laboratories Inc., CA, USA).

### Mutant isolation

The p416GPD-Pc-eRF3c DNA was mutagenized by incubation with 0.4 M hydroxylamine at pH 6.0 for 20 h at 37°C, or by the error-prone PCR method (41). Then, the mutagenized plasmid mixture was introduced into the eRF3ts strain (YK21–02) or the tet-OFF eRF3 strain (Y40), and the transformants were incubated on SC−Ura plates, under restrictive conditions, at 37°C, or in the presence of 7.5 μg/ml doxycycline, respectively. Plasmids from viable colonies were isolated and sequenced.

The p414GPD-Sc-eRF1 DNA was mutagenized by the error-prone PCR method. The mutagenized plasmid mixture was introduced into the double tet-OFF strain (Y138) together with p416-Pc-eRF3c. Then, the strain was incubated on SC−Ura−Trp plates containing 7.5 μg/ml doxycycline. Plasmids from viable colonies were isolated and sequenced. These selections were repeated until redundancy of the mutated positions among the singly or multiply mutated genes was observed.

### Stop codon read-through assay

The dual-luciferase-based translational read-through assay system was first reported by Grentzmann *et al.* (42), and, based there on, the eRF1 read-through assay, using strains with an eRF1ts (*sal4–2*) background and chromosomally integrated dual-luciferase reporter, was subsequently developed (24). Similar eRF3 read-through assay strains with an eRF3ts (*gst1–1*) background were constructed here. Assay strains harboring wild-type or mutant eRF expression plasmids were liquid-cultured in plasmid-selection medium at a permissive temperature (30°C), up to mid-log phase, and were then transferred to a non-permissive temperature, i.e. 37°C, for several hours, as indicated in the Results section. The cells were harvested, suspended in sample buffer and cell lysates were then prepared by vigorous shaking in the presence of glass beads using a FastPrep 24 instrument (MP-Biomedicals). Then, the lysates were applied to a dual-luciferase assay system according to the manufacturer's instructions (Promega, Madison, WI, USA), using a GloMax™ 96 Microplate Luminometer (Promega). Percent read-through was determined by the ratio of the firefly/renilla luciferase measurement of stop codons to that of the UGG sense codon-containing strain.

## RESULTS

### eRF3 from *P. carinii* does not complement depletion of *S. cerevisiae* eRF3

**Figure 1.**
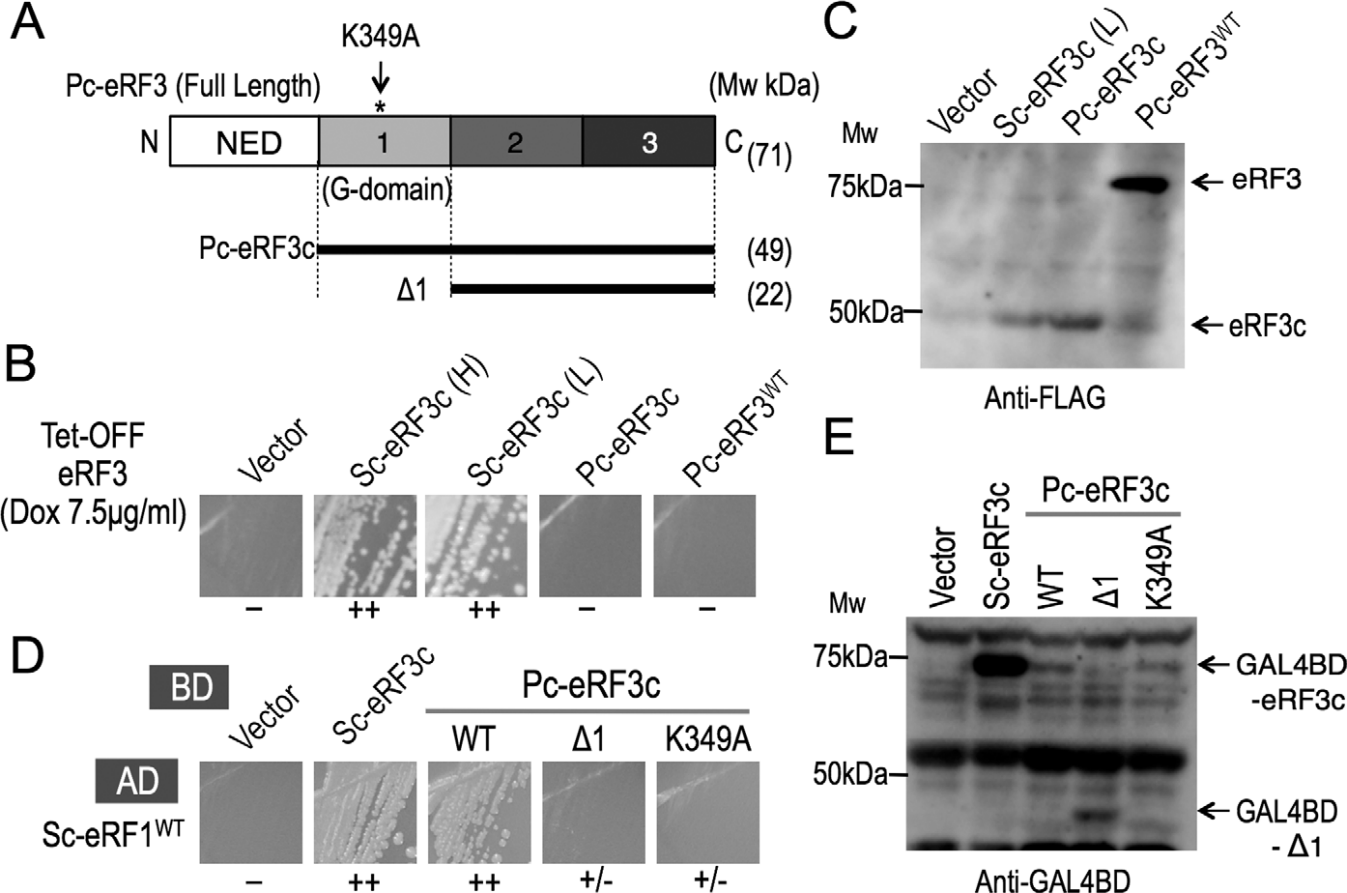
Characterization of Pc-eRF3 in budding yeast, *S. cerevisiae.* (A) Schematic drawing of the domain structure of eRF3 and its variants investigated in this study. eRF3 is composed of an N-terminal region (NED) and domains 1, 2 and 3, where domain 1 is the GTP-binding domain (G-domain), containing conserved motifs. Pc-eRF3 variants include Δ1, which lacks the G-domain, and K349A, which is a GTP-binding site mutation, within a conserved motif. (B) Complementation analysis of a tet-OFF eRF3 strain of *S. cerevisiae* (Y40). The p416GPD vector, p416GPD-Sc-eRF3c (Sc-eRF3c(H), here ‘H’ denotes high expression), p416CYC-Sc-eRF3c (Sc-eRF3c(L) here ‘L’ denotes low expression), p416GPD-Pc-eRF3c, p416GPD-full-length-Pc-eRF3, were introduced into Y40, and growth of transformants was examined in the presence of 7.5 μg/ml doxycycline. (−: no growth; +: weak growth; ++: good growth.) (C) Cellular expression of the N-terminally FLAG-tagged eRF3 variants by western blot analysis. Vector (p416GPD-Flag) and its variants harboring Flag-tagged Sc-eRF3c (p416CYC-Flag-Sc-eRF3c), Flag-tagged Pc-eRF3c (p416GPD-Flag-Pc-eRF3c) or Flag-tagged Pc-eRF3 (p416GPD-Flag-full-length-Pc-eRF3) were introduced into Y40 and cell lysates were prepared and analyzed as described in Materials and Methods, using the anti-FLAG monoclonal antibody. (D) Yeast two-hybrid analysis between Sc-eRF1 and Pc-eRF3s. Binding domain vector (pGBT9) and its variants, pGBT9-Sc-eRF3c, pGBT9-Pc-eRF3c^wt^, pGBT9-Pc-eRF3Δ1 and pGBT9-Pc-eRF3c-K349A, were co-introduced into the AH109 strain along with the activation domain fusion pGAD424-Sc-eRF1, and growth of transformants were examined on plates lacking histidine, as an indicator of interaction. (−: no binding; +/− slight binding; +: weak binding; ++: good binding.) (E) Cellular expression of the eRF3 variants fused to the binding domain in the AH109 strain. Cell lysates from the transformants in (D) were analyzed by western blotting with the anti-GAL4BD antibody.

Similar to other eRF3 orthologs, the eRF3 derived from *P. carinii* (Pc-eRF3) contains a less-conserved N-terminal region (1,9,18), which is thought to be dispensable for the eRF3 (*SUP35*) defective yeast cell growth complementation (1,43). Thus, two types of expression plasmids for full-length Pc-eRF3 and N-terminally truncated minimal Pc-eRF3, hereafter designated as Pc-eRF3c, were constructed for the activity test (Figure 1A) using an expression vector with a strong GPD promoter (p416GPD). Expression plasmids were introduced into the conditional-lethal eRF3 *S. cerevisiae* strain, Y40, in which the endogenous eRF3 promoter is replaced with the tet-OFF promoter, which can be deactivated in the presence of doxycycline. None of the Pc-eRF3 transformant colonies was able to grow when streaked on plates in the presence of the tetracycline analog, doxycycline (7.5 μg/ml). In contrast, the control transformants expressing the N-terminally truncated minimal wild type yeast eRF3 (hereafter designated as Sc-eRF3c), either under control of the GPD promoter (H), ‘H’ for high expression, or the weak CYC promoter (L), ‘L’ for low expression, complemented the defective growth (Figure 1B).

The FLAG-tagged variants of Pc-eRF3s as well as Sc-eRF3c were detected in the cell lysates by western blotting, revealing that the expression levels of Pc-eRF3s in the assay strains are sufficiently comparable to that of low expression Sc-eRF3c (L), which complements cell growth of Y40 (Figure 1C). Unlike other eRF3 orthologs that have been tested for yeast cell growth complementation to date, Pc-eRF3 was uniquely do not function with the *S. cerevisiae* translation termination machinery. This finding prompted us to attempt genetic analyses using Pc-eRF3 to elucidate the functional interplay among eukaryotic release factors (eRFs) and the ribosome during translation termination.

The binding between eRF1 and eRF3 has been extensively studied as a unique feature of eukaryotic translation termination factors and has recently been shown to be common in archaea; in contrast, class I and the class II release factors do not exhibit any direct binding in prokaryotes. Structural studies have revealed that class II release factors bind to class I release factors through contact sites located in three distinctive structural domains of eRF3, domains 1, 2 and 3 (22,24); this is quite similar to tRNA binding to EF-Tu/EF1α. On the other hand, mutational studies have revealed that (i) the principal binding is observed with the Δdomain1 variants of eRF3, which completely lack the conserved GTP-binding motifs in domain 1 (10,44), and that (ii) there are strong correlations between translation termination activity and binding efficiency (11,22).

Considering that defective binding to eRF1 could underlie the result of the yeast complementation test, binding between Pc-eRF3 and Sc-RF1 was examined by yeast two-hybrid analysis. Pc-eRF3c as well as its Δdomain1 variant (Δ1), fused to GAL4BD in pGBT9, and Sc-eRF1, fused to the GAL4-activation domain (GAL4AD) in pGAD424, were co-introduced into the AH109 reporter strain and its growth was then evaluated on assay plates lacking histidine. Consequently, Sc-eRF1 was shown to bind to Pc-eRF3c and to Sc-eRF3c equally well (Figure 1D). However, unlike other eRF3 orthologs reported to date, the deletion of domain 1 in the Δ1 construct of Pc-eRF3 did not exhibit binding to Sc-eRF1 (Figure 1D). Furthermore, introduction of a mutation into a conserved GTP-binding motif of Pc-eRF3c, K349A, resulted in a marked reduction of binding in the yeast two-hybrid assay (Figure 1D). The expression levels of the eRF3 fusion proteins were confirmed by western blot analyses using an anti-GAL4BD antibody (Figure 1E). These results clearly indicated that Pc-eRF3c is distinct from Sc-eRF3 and that Pc-eRF3c binding to Sc-eRF1 is highly dependent on the GTP-binding motif of domain 1.

Recent high-resolution X-ray structural analyses of the archaeal aRF1•aEF1α•GTP complex (and of its homolog), which should be functionally equivalent to the eRF1•eRF3•GTP complex in eukaryotes, clearly demonstrated the involvement of domain 1 in ternary complex formation, suggesting exact structural and functional mimicry between eRF1•eRF3•GTP and tRNA•EF1α•GTP complexes (25,26). Accordingly, it was predicted that a conformational change in the eRF1•eRF3•GTP complex would occur in the ribosome, as it does in homologous complexes (45). However, the detailed molecular mechanism by which the eRF1/eRF3 complex decodes stop codons on the ribosome is poorly understood. Taken together, we deduced that Pc-eRF3 could be defective in GTP-switching cooperativity with the yeast apparatus. Therefore, we attempted further genetic analyses of the heterologous eRF complex (Sc-eRF1/Pc-eRF3) to elucidate the molecular details underlying eRF1/eRF3 cooperativity.

### Pc-eRF3c mutations that restore growth of conditional-lethal eRF3 (*SUP35*) *S. cerevisiae* strains

**Figure 2.**
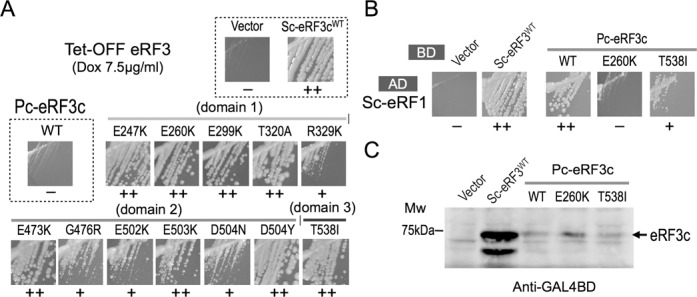
Isolation and analyses of Pc-eRF3 mutations. (A) Growth of tet-OFF eRF3 strains that had been transformed with the Pc-eRF3-mutant plasmids, in the presence of 7.5 μg/ml doxycycline, is shown. (B) Yeast two-hybrid binding assay of Pc-eRF3c mutants against Sc-eRF1. Binding domain vector pGBT9 alone, or fused to Sc-eRF3c, Pc-eRF3c^wt^, or its mutant genes were introduced into the AH109 strain together with pGAD424-Sc-eRF1, and growth of the transformants were examined on selection plates. The growth of two notable mutants, E260K and T538I, are shown in this figure. A complete data set, including data of all mutations, is provided in Supplementary Figure S4. (−: no binding; +: weak binding; ++: good binding.) (C) Cellular expression of eRF3 variants fused to the binding domain in the AH109 strain. Cell lysates from the transformants in (B) were analyzed by western blotting with the anti-GAL4BD antibody.

Pc-eRF3c in the p416GPD vector was randomly mutagenized by hydroxylamine treatment or by error-prone PCR, to obtain Pc-eRF3c mutant libraries. The libraries were introduced into conditional lethal eRF3 yeast strains, either YK21–02 (eRF3ts) or Y40 (tet-OFF eRF3), and viable transformants were selected under restrictive conditions to obtain Pc-eRF3c mutations that restore cell growth in presence of Pc-eRF3 as the sole source of eRF3. Consequently, 12 independent missense mutations were isolated involving 11 amino acid positions located throughout the 3 domains of Pc-eRF3c (Table 1, Figures 2A and 6A). Remarkably, seven mutations alter amino acids conserved in Sc-eRF3 (Supplementary Figure S1). Intriguingly, two of the mutations, E260K and T538I, were located in the region interfacing with Sc-eRF1, as deduced from the structure of the homologous complex aRF1•aEF1α•GTP (PDB ID: 3VMF) (Figure 6A and C). The other mutations altered amino acids located on the surfaces not involved in the binding-interface of the eRF1•eRF3 complex, as deduced from the structures of homologous complexes (26). G-domain mutation sites, E247, T320 and R329, are likely to interact with the 25S rRNA of the ribosome, although E247K may also affect the binding of GTP. Only the E299K mutation in domain 1 maps to the intramolecular contact site with domain 3. Moreover, the five mutation sites in domain 2 are located close to reported 18S rRNA interaction sites.

**Figure 3.**
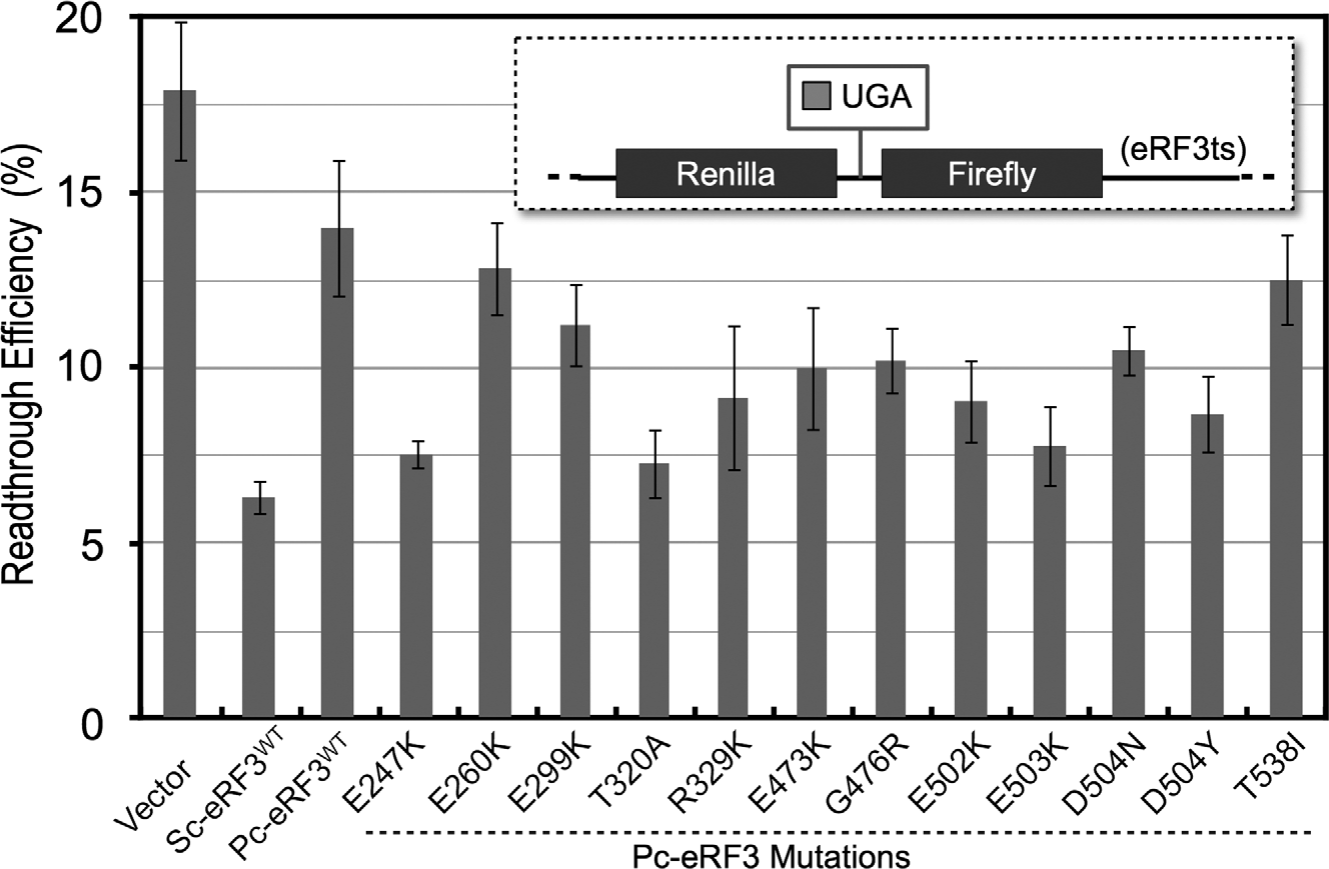
Read-through efficiency of Pc-eRF3c mutants. Transformants of the eRF3ts read-through assay strain (Y133 for UGA, Y134 for UGG), harboring the expression vector p416GPD (blank), p416GPD−Sc-eRF3c, p416GPD−Pc-eRF3c^wt^ or its mutants, were applied in a dual-luciferase assay. Read-through efficiency was calculated and data are shown as the ratio to the UGG-containing control (See Materials and Methods and references therein for detail).

**Figure 4.**
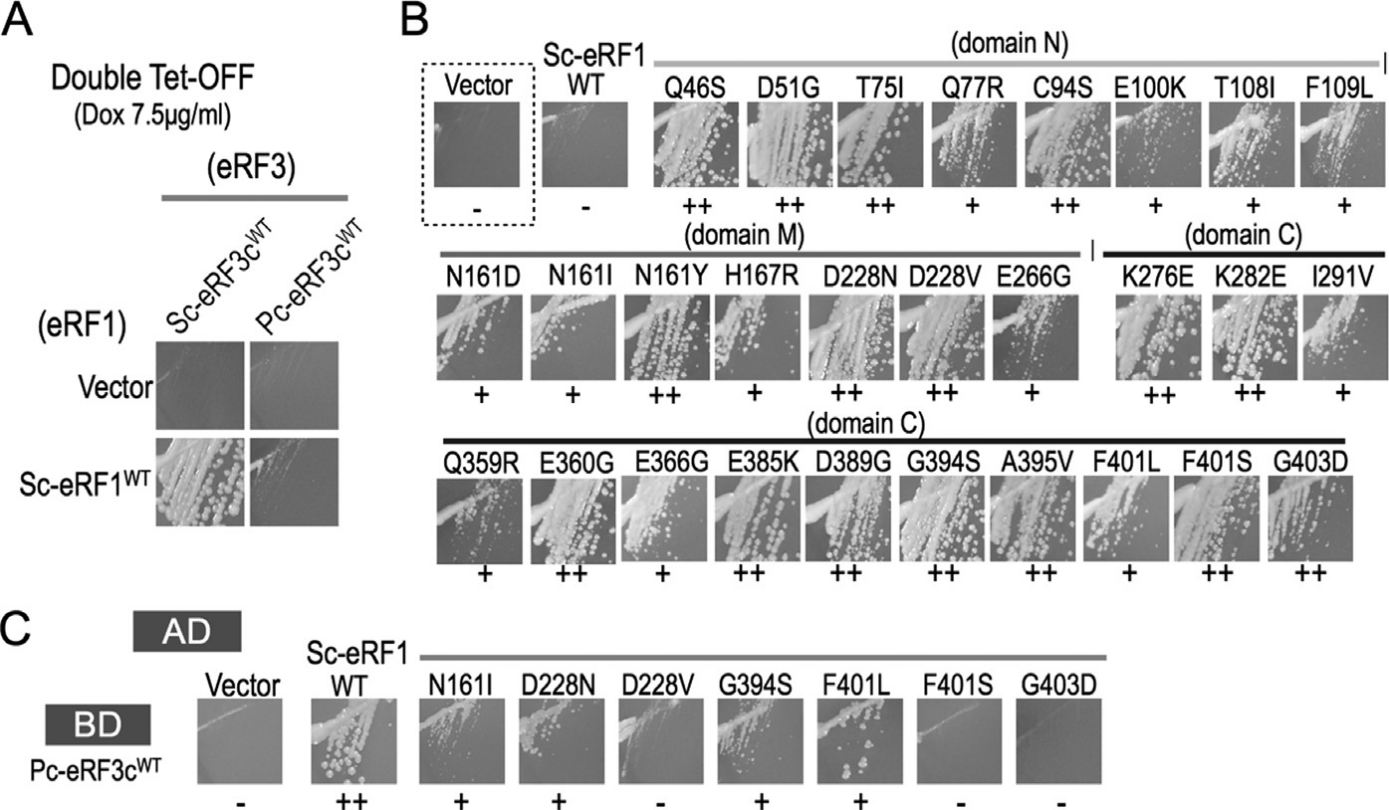
Isolation and analyses of Sc-eRF1 mutations. (A) Complementation analysis of the double tet-OFF strain of *S. cerevisiae* (Y138). The P414GPD vector and p414GPD-Sc-eRF1 (SC-eRF1^WT^) were introduced into Y138, in combination with p416GPD-Sc-eRF3c^wt^ (Sc-eRF3 ^WT^) or p416GPD-Pc-eRF3c^wt^ (Pc-eRF3c ^WT^), and growth of the transformants were examined in the presence of 7.5 μg/ml doxycycline. (−: no growth; +: weak growth; ++: good growth.) (B) Growth of the double tet-OFF strain that had been transformed with the Sc-eRF1 mutant plasmids, together with Pc-eRF3c ^wt^ plasmids, in the presence of 7.5 μg/ml doxycycline, is shown. (C) Yeast two-hybrid binding assay of Sc-eRF1 mutants against Pc-eRF3c ^wt^. The activation domain vector pGAD424 and its variants, harboring Sc-eRF1 ^wt^ and its mutant genes, were introduced into the AH109 strain together with pGBP9-Pc-eRF3c^wt^, and the growth of transformants examined for growth on selection plates. The growth of seven notable mutants, N161I, D228N, D228V, G394S, F401L, F401S and G403D, are shown in this figure. A complete data set, including those of all mutations, is provided in Supplementary Figure S6.

**Figure 5.**
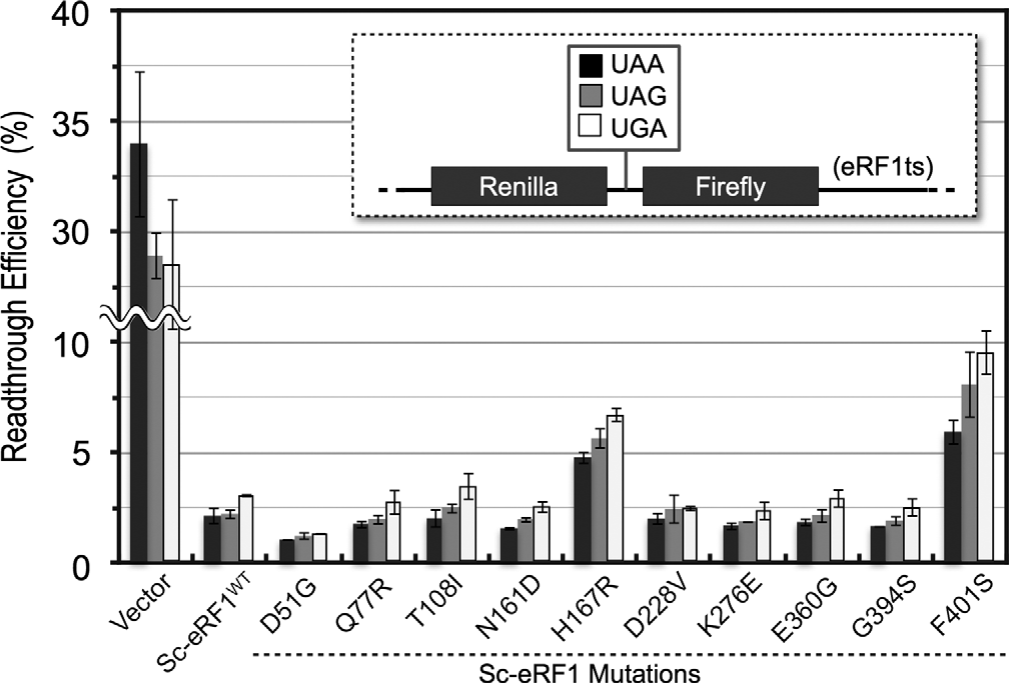
Read-through efficiency of Sc-eRF1 mutants. Transformants of the eRF1ts read-through assay strains (S13-I01, S13-I03, S13-I05 and S13-I07 for UAA, UAG, UGA and UGG, respectively) harboring the expression vector p416CYC (blank), p416CYC−Sc-eRF1 and its mutants were applied for dual-luciferase assay. Read-through efficiency was calculated and shown as a ratio to UGG containing control (See Materials and Methods and references therein for detail).

**Figure 6.**
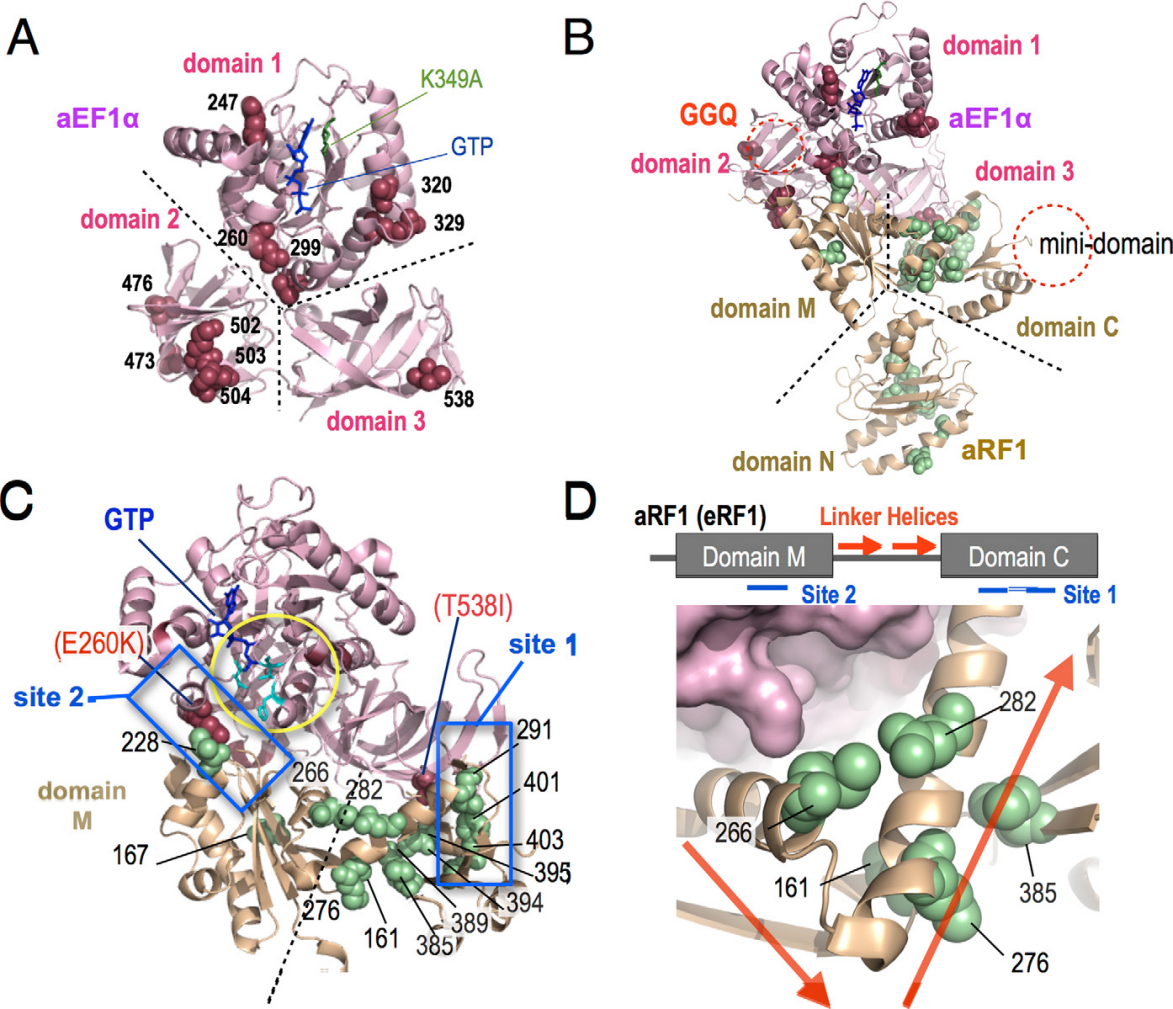
Location of mutations on crystal structures. (A) The sites of Pc-eRF3c mutations identified in this study are shown by spheres, in raspberry, on the aEF1α crystal structure (from the aEF1α•aRF1•GTP complex [3VMF]), according to the homology alignment (Supplementary Figure S1). K349A, a putative GTP-binding site, is shown in green. Note that all residues are indicated in Pc-eRF3 numbering. Structural domains 1, 2 and 3 of aEF1α (as well as eRF3s) that correspond to those in Figure 1A. (B) Sc-eRF1 mutation sites are shown as green spheres, in addition to the mutations in (A), on the structure of aRF1 within the aEF1α•aRF1•GTP complex, based on homology alignment. Mutation sites in Figures 6 and 7 are shown in Sc-eRF1 numbering. Putative motif regions are indicated as follows: GGQ, corresponding to CCA-end of tRNA (which is omitted in the process of crystallization); the mini-domain, a eukaryotic eRF1-specific region that interacts with the 40S beak. Corresponding structural domains N, M and C of aRF1 (as well as eRF1s) are also indicated. (C) Close-up view of the eRF1/eRF3 interaction regions. Sc-eRF1 mutations are shown as in (B). The two major direct interaction sites, site 1 and site 2, are boxed in blue. (site 1: classical binding site at the C-terminals; site 2: G-domain interaction site). Critical interface mutations in eRF3, E260K and T538I, are shown as spheres. Catalytically important His84 and gate residues are shown using cyan stick models. (D) Further close-up view around the linker helices regions of eRF1. (Upper) Schematic drawing of linker helices regions of aRF1 (eRF1). In the aEF1α•aRF1•GTP complex, linker helices (red arrows) make a compact bend, connecting domains M and C. The two major binding interfaces, site 1 and site 2, are indicated. (Lower) Clustering of the three mutations in the linker helices region (indicated with arrows, as above; at residues 266, 276 and 282) and two distinct mutations in domain M (residue 161) and domain C (residue 385) is shown as green spheres. All 3D-structures were visualized using the PyMol Molecular Graphics System (Schrödinger, LLC.).

**Table 1.** Summary of Pc-eRF3c mutations

Domain	Mutation	No. of isolates*	Complementation		Y2H*** with Sc-eRF1	Corresponding residue^§^ in aEF1α (Ap)
			eRF3 ts **	tet-OFF eRF3**		
	wild type		−	−	++	
Domain 1	E247K	3	++	++	++	E51
	E260K	1	+	++	−	E64
	E299K	3	++	++	++	T103
	T320A	4	++	++	++	A124
	R329K	1	+	+	++	R133
Domain 2	E473K	2	++	++	++	E275
	G476R	1	+	+	++	S278
	E502K	2	+	+	++	K304
	E503K	1	++	++	++	S305
	D504N	1	+	+	++	D306
	D504Y	1	++	++	++	D306
Domain 3	T538I	2	+	++	+	T340

*Number of times that the mutation was isolated independently during screening.

**Complementation level of eRF3ts strain and tet-OFF eRF3 strain are indicated; −: no growth; +: weak growth; ++: good growth.

***Growth level in yeast two-hybrid (Y2H) binding assay with Sc-eRF1 are indicated; −: no binding; +: weak binding; ++: good binding.

§ Residues in aEF1α corresponding to the Pc-eRF3 mutation sites, according to homology alignment.

### Pc-eRF3c mutants restore termination efficiency in a stop codon read-through assay

The translation termination-stimulating activity of wild-type Pc-eRF3c (hereafter referred to as Pc-eRF3c^wt^) and mutant Pc-eRF3cs in the cell were examined using stop codon read-through assays. The dual-luciferase constructs containing the UGA stop codon or the UGG sense codon between two different luciferase genes, encoding renilla and firefly luciferase, were stably integrated into the chromosome of the temperature-sensitive eRF3 (eRF3ts) strain (Materials and Methods). The assay strains transformed with each of the eRF3 expression vectors were liquid-cultured, transferred to a restrictive temperature (37°C), and after 4 h, the luciferase activity was measured to calculate the read-through efficiency. Read-through efficiency was shown as a ratio to the control strain containing a UGG (Trp) codon, instead of the UGA codon. As expected from the growth complementation assay, the Pc-eRF3c^wt^ exhibited high read-through efficiency compared with wild-type Sc-eRF3c, i.e. is severely defective in translation termination, and showed only slight decrease in read-through efficiency compared with the control vector. On the other hand, most of the Pc-eRF3c mutations exhibited a significant decrease in read-through (Figure 3). The read-through efficiencies of the variant Pc-eRF3s were mostly consistent with the growth complementation of *eRF3ts* strain (Table 1), clearly supporting reasonable genetic selection. However, two of the mutants in which the eRF1-binding interface of eRF3 was affected, E260K and T538I, showed less evident recovery of translation termination activity, despite their moderate growth complementation (Table 1, Supplementary Figure S3).

### Reduced eRF1 binding by the binding interface mutations of Pc-eRF3c, E260K and T538I

The eRF3 mutants obtained were also examined for their ability to bind to Sc-eRF1, in order to assess the correlation between eRF1/eRF3 binding and translation termination efficiency in the cell. Interestingly, the binding interface mutants of Pc-eRF3c, E260K and T538I, exhibited strikingly reduced binding to Sc-eRF1 (Figure 2B), while the other Pc-eRF3c mutations showed binding comparable to that of Pc-eRF3c^wt^ (Supplementary Figure S4). The reduced binding of Pc-eRF3 E260K and T538I mutants to Sc-eRF1 accounts for the inefficient recovery of translation termination activity mentioned above, since inactivated eRF3ts proteins at 37°C, in the read-through assay strains, more efficiently compete with these Pc-eRF3 mutations for Sc-eRF1 binding. The better growth results for Pc-eRF3 transformants harboring E260K or T538I in the tet-OFF eRF3 strain, as compared to those of eRF3ts strains, are consistent with this idea (Table 1). The cellular expression levels of Pc-eRF3cs fused to GAL4BD were examined by western blotting analysis with an anti-GAL4BD antibody; this confirmed that the mutant proteins were expressed at levels comparable to those of Pc-eRF3c^wt^ (Figure 2C).

### Isolation of Sc-eRF1 mutations co-operating with Pc-eRF3c^wt^

Taking a reverse approach, we then attempted to isolate Sc-eRF1 mutations that restore cell growth in the presence of wild-type Pc-eRF3c (Pc-eRF3c^wt^) as the sole source of eRF3 in *S. cerevisiae*, in order to elucidate the co-operativity of the eRF1•eRF3 complex from the eRF1 perspective. To this end, we have constructed an assay strain named ‘double tet-OFF’ (Y138) in which the endogenous promoters of eRF1 and eRF3 genes on chromosomes were replaced with the tet-OFF promoter and the expression of endogenous eRF1 in addition to eRF3 can be repressed by addition of tetracycline (or its derivative, doxycycline). Thus, only successful combinations of heterologous or homogeneous release factors, eRF1 and eRF3, introduced via plasmids, can support the growth of such a strain under non-permissive conditions.

To confirm that the strain functions as expected, the wild type and the mutant Pc- or Sc-eRF3s, in combination with Sc-eRF1, were introduced into the strain under permissive conditions (0 μg/ml doxycycline) and cell growth was then monitored under non-permissive conditions (7.5 μg/ml doxycycline). Consistently, a homogeneous Sc-eRF pair, as well as Sc-eRF1 paired with the Pc-eRF3c mutations, suppressed cell growth, while a combination of Pc-eRF3c^wt^ and Sc-eRF1 did not (Figure 4A and Supplementary Figure S5).

Then, using the double tet-OFF strain, Sc-eRF1 mutants that could support cell growth under restrictive conditions in the presence of Pc-eRF3c^wt^ were isolated. The randomly mutagenized Sc-eRF1 library and the Pc-eRF3c^wt^ plasmid were co-introduced into the double tet-OFF strain and the mutant Sc-eRF1 plasmids were isolated from transformants that were viable in the presence of doxycycline. Consequently, 28 independent mutations were isolated in total (Table 2 and Figure 4B).

**Table 2.** Summary of Sc-eRF1 mutations

Domain	Mutation	No. of isolates*	Functionality** with Pc-eRF3c^wt^	Y2H*** with Pc-eRF3c^wt^	Corresponding residue* in aRF1
	wild type		−	++	
Domain N	Q46S	1	++	++	M49
	D51G	4	++	++	Q54
	T75I	1	++	++	A78
	Q77R	1	+	++	D80
	C94S	1	++	++	C97
	E100K	1	+	++	−
	T108I	1	+	++	C108
	F109L	1	+	++	F109
Domain M	N161D	2	+	++	A161
	N161I	1	+	+	A161
	N161Y	2	++	++	A161
	H167R	1	+	++	K167
	D228N	1	++	+	L229
	D228V	1	++	−	L229
	E266G	1	+	++	M268
Domain C	K276E	4	++	++	M278
	K282E	1	++	++	N284
	I291V	1	+	++	L293
	Q359R	1	+	++	−
	E360G	6	++	++	−
	E366G	1	+	++	−
	E385K	2	++	++	I343
	D389G	2	++	++	E347
	G394S	1	++	+	A352
	A395V	1	++	++	E353
	F401L	1	+	+	F359
	F401S	2	++	−	F359
	G403D	1	++	−	G361

*Number of times that the mutation was isolated independently during screening.

**Co-functional level with Pc-eRF3c^wt^ in double tet-OFF strain were indicated; −: no growth; +: weak growth; ++: good growth.

***Growth level in yeast two-hybrid (Y2H) binding assay with Pc-eRF3cwt are indicated; −: no binding; +: weak binding; ++: good binding.

§Residues in aRF1 corresponding to the Sc-eRF1 mutation sites, according to homology alignment.

**Figure 7.**
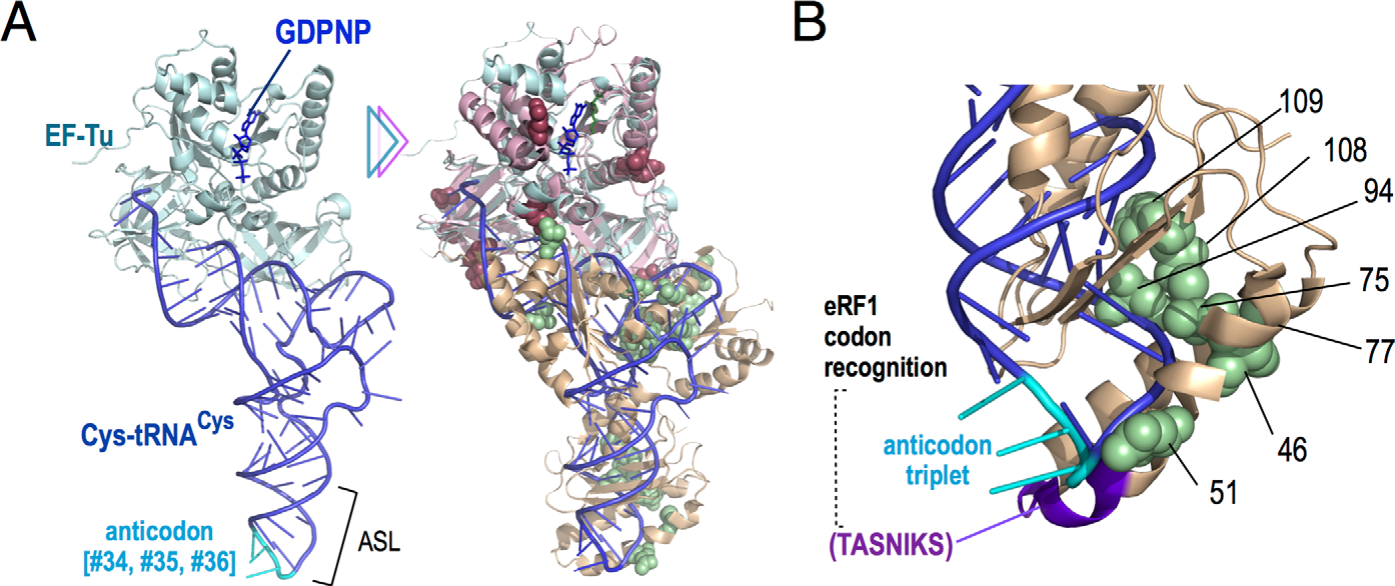
Superposition of the crystal structures of EF-Tu•tRNA^Cys^•GDPNP and aEF1α•aRF1•GTP with mutations identified in this study (A). (Left) X-ray crystal structure of EF-Tu•tRNA^Cys^•GDPNP complex (1B23) and the tRNA anticodon is shown in cyan. (Right) Superimposition of the EF-Tu•tRNA^Cys^•GDPNP complex (1B23) and the aEF1α•aRF1•GTP complex (3VMF), based on G-protein homology, with mutations of eRF1 and eRF3, as shown in Figure 6B. (B) Close-up view of the eRF1-ASL mutations on the superimposed model (in Sc numbering). eRF1-ASL mutations (shown in green spheres) cluster on one side of the model, opposite to the TASNIKS motif and the tRNA anticodon triplet. All 3D structures were visualized using the PyMol Molecular Graphics System.

In the interface region, 7 mutations affecting 4 amino acid positions in domain M and 13 affecting 12 amino acid positions in domain C, were isolated (Figure 6B and C). Interestingly, eight mutations were located in domain N, which is not involved in the binding interface with eRF3. (Please note that domains N, M and C correspond to the A, B and C domains, respectively, of archaeal RF1 in the previously reported structure). The mutations in domain N are clustered at the tip of the region opposite the decoding regions reported previously (Figure 7B) (5,22,46). The domain M mutation site D228 faces the G-domain switch region of eRF3 in the structure of the complex, and potentially makes direct contact with E260 (Pc numbering) of eRF3. Conversely, a mutation at E260 of Pc-eRF3 had been isolated in the earlier Pc-eRF3 selection (Figure 6C). H167R mutation in domain M was located at the non-eRF3 side surface of domain M and presumably makes contact with the ribosomal S23 protein (correspond to prokaryotic S12) on the ribosome (28). Three mutations, E266, K276 and K282, were located within the linker helices region, which are thought to connect flexibly to two structural domains of eRF1, namely, domains M and C, and were modeled to play crucial roles in coupling with the GTPase switch of eRF3 (Figure 6D) (24). Two mutations, N161 and E385, in domains M and C, respectively, seemed to make close contact with the linker helices in the archaeal complex (Figure 6D). Furthermore, at position N161, three different missense mutations were obtained, suggesting that these contacts are essential to the function of the complex. The structural significances of these mutations will be discussed later. The remaining domain C mutations are clustered in two discrete locations. One is the historically well-studied major binding site with domain 3 of eRF3, named site 1. The other, intriguingly, involves the extra region that is unique to eukaryotes and has not been studied to date. Thus, mutations located in this region may explain an as-yet-unknown regulatory role unique to eukaryotes.

### Binding of Sc-eRF1 mutants to Pc-eRF3c^wt^ in the two-hybrid assay

Yeast two-hybrid binding analysis was conducted for the Sc-eRF1 mutations and Pc-eRF3c^wt^. Intriguingly, the Sc-eRF1 mutations N161I, D228N, D228V, G394S, F401L, F401S and G403D exhibited reduced binding to Pc-eRF3c^wt^ (Figure 4C and Table 2), while others did not affect the binding (Supplementary Figure S6). Those mutations showing reduced binding are located either on the surface regions involved in interaction with eRF3, or among the ‘linker helices’ region. Notably, the highly conserved amino acid residue F401 (*S. cerevisiae* numbering, F405 in *S. pombe*; Supplementary Figure S2) on the contact regions of domain C are reported to be crucial for binding as well as for translation termination (22). One of the three isolocus mutants, N161I, clearly exhibited reduced binding with eRF3.

### Effect of Sc-eRF1 mutations on translation termination

To evaluate Sc-eRF1 mutations, a dual-luciferase read-through assay was again conducted in a yeast strain with Sc-eRF1ts (*SUP45, sal4–2*) and wild-type Sc-eRF3 (*SUP35^+^*) background (22,47). Ten of 28 mutations were selected as representative mutations and were evaluated using this assay (Figure 5). Intriguingly, two of the mutations, H167R and F401S, exhibited reduced termination efficiency *in vivo* (Figure 5). This was consistent with the concept that the Sc-eRF1 mutations were selected for growth complementation of the double tet-OFF strain with the exogenous Pc-eRF3, but not for stronger termination with endogenous Sc-eRF3. The domain N mutations apparently did not affect termination efficiency, except for the D51G mutation, which markedly enhanced termination activity (Figure 5). A previous report described a series of Sc-eRF1 mutations screened for their stronger termination activity at leaky stop codons; these mutated proteins were named ‘hyperactive eRF1s’ (48). Intriguingly, those proteins included mutations at the same amino acid positions (E46, D51, F109, N161 and E360) as in this study. Among them, an identical mutation, i.e. E360G, exhibited a 3-fold increase in termination efficiency in the previous study, but exhibited comparable efficiency to that of wild-type protein in this study. Although the inconsistent effects of the E360G mutation could not be explained in both studies due to the differences in the assay systems employed, it is worth noting that the same amino acid residues were selected by two different sets of criteria for eRF activity. The domain N mutations in this study hardly affected the codon specificities for the three different stop codons, UAA, UAG and UGA (Figure 5). Thus, these results from eRF1 analysis confirmed that the heterodimeric binding and termination activities of eRFs do not always correlate well. This will be discussed later.

## DISCUSSION

Our yeast-based genetic analyses using Pc-eRF3 revealed novel aspects of the mechanism by which the eRF1•eRF3 complex functions in the process of translation termination on the ribosome. In addition to the classical binding sites between eRF1 and eRF3, our results suggest the participation of novel sites both in eRF1 and eRF3, which are involved in making appropriate contact not only between eRFs, but also with various ribosomal sites, in this process.

### Binding and co-operativity between eRFs

Binding interface mutations that are supposed to be crucial for functional co-operation of eRFs were identified at two pairs of loci in Pc-eRF3 and Sc-eRF1: one involves domain M of eRF1 versus the GTP-binding domain of eRF3 (site 2, Figure 6C); the other is domain C of eRF1 versus domain 3 of eRF3 (site 1, Figure 6C). The interface mutations, E260K, in the G-domain of eRF3, and D228N and D228V of eRF1 are included within site 2, as predicted in the aEF1α•aRF1 complex (24). Importantly, the E64A mutation in aEF1α (corresponding to E260K in Pc-eRF3) is reported to cause loss of binding activity in the aEF1α•aRF1 complex (26); thus, this site is crucial for functional co-operativity as well as for binding, in both eRF complexes. In addition, the D228N and D228V mutations in eRF1 reduced its binding to eRF3, also indicating the important role of site 2 interactions in eukaryotes. Residue E260 in the G-domain is close to the conserved V20, I60, and H84 residues of EF-Tu (*E. coli* numbering) that are known to be necessary for GTPase activity (Figure 6C) (49,50), suggesting close functional association between site 2 binding and GTPase activation, which has been implicated previously (22).

A series of mutations at T538 of Pc-eRF3 and at I291, G394, A395, F401 and G403 of Sc-eRF1 are located in the classical site 1 interface region, which is composed of domain C of eRF1 and domain 3 of eRF3 (22). Consistently, our yeast two-hybrid analysis revealed that the mutations T538I in Pc-eRF3 and F401L, F401S and G403D in Sc-eRF1 markedly reduced binding of these proteins, as partly observed in previous analyses (22). Intriguingly, unlike in the archaeal aEF1α•aRF1•GTP ternary complex, incorporation of T538 (T340 in ap-aEF1α) in the contact site has not been observed in the previously reported eukaryotic complex structures that lack domain 1 of eRF3, which contains GTP-binding motifs. Those results strongly suggest that integrity of this interface region is necessary for functional co-operation between eRFs, and that this is assisted by allosteric changes among the individual eRF1 and eRF3 domains upon guanine nucleotide binding; such correlations have not been observed in previous genetic or structural analyses of eukaryotic release factors. In agreement with this speculation, the G domain variants of Pc-eRF3, Δdomain 1 and K349A, exhibited severe loss-of-function in yeast two-hybrid binding assays (Figure 1C).

In addition to the site 1 and site 2 mutations, mutations in the linker helices, E266G, K276E and K282E, and mutations in the adjacent domain, N161D, N161I, N161Y and E385K, were isolated (Figure 6C and D). Although the locations of these mutations are mapped apart from the contact sites for binding, the resulting phenotypes were comparable. Interestingly, in the archaeal complex, the affected residues in the adjacent domains B and C, N161 and E385, respectively, make close contact with the linker helices residues (Figure 6D), presumably, to restrict the flexibility between domains B and C. Therefore, those mutations could strongly affect binding coordination of domains B and C of eRF1, to ensure integrity of the interface with eRF3. In accordance with this hypothesis, one of linker helices mutations, N161I, led to a reduction in eRF3-binding. This is the first evidence that remote site mutations in eRF1 can affect eRF3-binding.

Pc-eRF3 mutations E260K and T538I as well as Sc-eRF1 mutations N161I, D228N, D228V, G394S, F401S and G403D exhibited reduced binding activity in yeast two-hybrid assays. These results indicated that formation of a tight complex between eRF1 and eRF3 through the binding sites, ‘site 1’ (between their C-terminal domains) and ‘site 2’ (between domain M of eRF1 and domain 1 of eRF3), was not prerequisite but rather harmful, suggesting that moderate binding might ensure efficiency in later steps of translation termination by eRFs on the ribosome. Furthermore, unexpectedly, the Sc-eRF1 mutations N167R and F401S conferred weaker translation termination activity in *S. cerevisiae*, i.e. with endogenous Sc-eRF3, in exchange for the suppression of Pc-eRF3 lethality. This suggests, at least in part, that the mutated Sc-eRF1/Pc-eRF3 complex might have bypassed its harmful effects by adjusting their binding configuration to be suitable for the subsequent reactions, such as codon recognition and catalysis of polypeptide chain release as well as GTP hydrolysis, on the ribosome. *In vitro* analyses of the elementary processes in translation termination should be performed to confirm this.

### Putative interactions with ribosomal subunits

Mutations in domain 1 of eRF3, E247, E299, T320 and R329, are distinguished from the contact areas for binding (Figure 6B) as seen in the complex model. It has been reported that the ribosomal sarcin–ricin loop (SRL), which is a conserved, essential loop structure of 25S rRNA that is located at the translational GTPase binding site, appears to be situated in the neighborhood of eRF3 residues E247, T320 and R329, as well as being close to the binding interface residue E260 in the pre-termination ribosomal complex model (30). Taking into account the high conservation and emerging importance of the ribosomal SRL and conservation of residue His84 among translational GTPases (50,51), those residues found to be mutated in this study are thought to form an external contact interface with the ribosome for appropriate GTPase activation.

In the eRF-specific regions of domain C in eRF1, i.e. the mini-domain that is not conserved in archaeal aRF1s, we identified a new class of mutations. The X-ray structure of this region has been left unsolved in the structure of apo-eRF1 as well as in structures of complexes with eRF3, due to the flexibility of this domain. The NMR solution structure of domain C was solved and the extra domain, termed the ‘mini-domain’, was reported to affect codon-specificity (52). According to the latest docking model-assisted cryo-EM study (30), three mutations (E285K, D389G and G394S) identified in this study are located in this ‘mini-domain’, which interacts with the ‘40S beak’, a structural protrusion of the 40S ribosomal subunit that is close to the entry site for translation factors, while the other domain C mutations probably interact with eRF3 or the P-stalk of the large 60S subunit. Taken together, those mutations are thought to affect the functional co-operation of the eRF1•eRF3 complex in a manner coupled to the GTPase-stimulating activity of the ribosome, and so on. It is interesting to speculate that the extra domain may facilitate self-binding of eRF1 on the ribosome, as occurs with bacterial class I release factors, since it is well known that eRF1 alone can catalyze stop codon-dependent peptide releasing reaction *in vitro*, in the absence of eRF3 (53).

As can be seen in the superimposed model (Figure 6C), among the mutations in domains M and C of eRF1, H167R is unique in that it is located distantly from the binding interface; it also caused reduced translation termination activity (Figure 5). In the X-ray structure of 70S ribosome•EF-Tu•tRNA complex, the tRNA in the ribosomal A-site is located such that it interacts with the ribosomal protein S12 (S23 of S*. cerevisiae*) at the acceptor-arm/D-stem junction (28), as well as the anticodon stem-loop (ASL) at the opposite end. It has been reported that the streptomycin-resistant phenotype caused by mutation of the bacterial S12 protein is negated by a mutation in EF-Tu, indicating co-operative signal relay of codon recognition to EF-Tu through the tRNA and the ribosome (54). Thus, according to tRNA mimicry-related geometry, it can be speculated that eRF1 could interact with S23 (the *S. cerevisiae* equivalent of S12) through the H167 region, although this should be examined further in future.

Since we have shown that, as compared to endogenous binding of eRFs, the binding of Sc-eRF1 to exogenous Pc-eRF3c is more dependent on the secondary binding site, although it also depends on the classical binding site, the defect(s) of Pc-eRF3 in yeast could be caused by an imbalance between the contributions of the two major interaction sites located at the interface with Sc-eRF1. Many of the mutations in both eRFs that suppress the defect(s) of Pc-eRF3 in yeast affected conserved amino acid residues (Supplementary Figures S1 and S2), and many of these mutations did not necessarily reside within interaction sites, but were located nearby (typically seen with eRF3 domain 2 mutations). Thus, the mutations seem to induce the conformational change necessary for GTPase activation, adjusting eRF1/eRF3 interaction in their vicinity; this links stop codon recognition to the GTPase switch.

### eRF1-ASL-like (eRF1-ASL) mutations

Domain N of eRF1, which contains the tip-like structure comparable to the ASL of tRNA, hereafter referred to as eRF1-ASL (eRF1 ASL-like), has been intensively studied in order to clarify the motifs and mechanisms involved in stop codon recognition and discrimination. Almost all of the known, well-studied, mutations that affect the specificity of stop codon recognition are localized on the side of the eRF1-ASL that would consistently face the stop codon bases in the ribosomal A site, as seen in the docking model structure of the aEF1α•aRF1•GTP complexed with the ribosome, as well as in the latest cryo-EM studies of the eRF3•eRF1 dimer complexed with the ribosome. However, the novel domain N mutations identified in this study clustered in a locus opposite to that previously noted (Figure 7A and B).

According to the superimposition of aEF1α•aRF1•GTP and EF-Tu•tRNA^Cys^•GDPNP, seven of eight eRF1-ASL mutations, except the eukaryote-specific E100R, are located along one side of the eRF1-ASL, corresponding to nucleotides 29–32 in the tEF-Tu•tRNA^Cys^•GDPNP structure (Figure 7A) (55). TASNICS and Y-C-F motifs (5,14,46,56), as well as other critical residues (22,57,58) responsible for codon selectivity, are apart from this region and consistently overlap with anticodon regions (nucleotides 34–36). In addition to the anticodon motif, other nucleotide positions of tRNAs, surrounding the anticodon, are known to modulate the decoding efficiency through nucleotide modification, structural interactions and so on (59–61). Intriguingly, many of the eRF1-ASL mutations identified in this study occurred at relatively conserved amino acid residues (Supplementary Figure S2). In the read-through assay of Sc-eRF1, two of the mutations, H167R and F401S, caused a marked reduction in translation termination efficiency at all three stop codons in *S. cerevisiae* (Figure 5), confirming that the mutations were not isolated simply because they generally enhanced termination activity. Only the D51G mutation, which is located most closely to the codon-recognition motifs, exhibited enhanced translation termination. Other eRF1-ASL mutations hardly affected translation termination at any of the three stop codons, and none of the eRF1-ASL mutations showed altered codon selectivity, as was seen in the previously noted mutations. These facts indicate that the mutations isolated by this strategy suppressed the defect of Pc-eRF3 in combination with Sc-eRF1 in yeast, because of certain conformational reassignments for functional co-operativity with Pc-eRF3, as well as with the ribosomal components in yeast. The eRF1-ASL mutations are thought to adjust the contact of the Sc-eRF1/Pc-eRF3 complex with other decoding components on the ribosome.

It has been reported that the anticodon stem region of the EF-Tu•tRNA^Cys^•GDPNP complex is conformationally distorted upon binding to the ribosomal A-site, and that this allows the whole complex to make precise contact at multiple functional sites in the large and small ribosomal subunits during decoding (62). This would explain why, in the Hirsch suppresser tRNA, mutations distant from the anticodon can still contribute to the distortion of tRNAs (63). Therefore, it is tempting to speculate that the residues mutated in the eRF1-ASL participate in a mechanism that mimics the dynamic structural rearrangement of tRNA on the ribosome, in order to orchestrate between the stop codon recognition mediated by eRF1 and the GTPase activation of eRF3.

Intriguingly, in the prokaryotic class I release factors (RF1, RF2), a series of charge-flip mutations of the relatively conserved residues that cluster within the ASL domains, adjacent to the decoding tripeptide motifs (PEP-anticodon motifs; PAT in RF1 for UAA, UAG, SPF in RF2 for UAA and UGA) were analyzed in our previous studies (64,65). The variant bacterial class I release factors are capable of polypeptide release at non-cognate stop codons, including sense codons, *in vitro*. Furthermore, those alterations also suppress the defects by certain types of dominant-lethal ASL mutations (64,65). It has been speculated, based on structural data, that correct positioning of the release factor domains is assisted by elaborate interactive networks among the mRNA, 16S rRNA, P-site tRNA and the ASL region of RFs, in addition to the direct codon-recognition motif (66). Interestingly, the eRF1-ASL mutations identified in this study could be sterically and phenotypically comparable to those bacterial mutations, although bacterial class I release factors exert their effects on the ribosome independently, and do not require translational GTPases, such as EF-Tu.

By genetic analyses, using a heterologous eRF3, this study revealed clues for novel aspects of the manner in which the eRF1/eRF3 complex mimics the tRNA/EF1α complex. It is likely that, due to their tRNA-mimicking strategy adopted in evolution, the eukaryotic translation termination complex eRF1 and eRF3 derived from heterologous species in previous reports exhibited high complementarity (22,38,44). However, in this study, we unexpectedly found that the wild-type Pc-eRF3 is a rare exception, in terms of its complementarity for eRF3 depletion in budding yeast. Genetic studies revealed that the defect of Pc-eRF3 in yeast could be rescued by multiple classes of single point mutations located in either of the eRFs; this yielded novel mechanistic insights into the molecular functions and interactions involved in the translation termination machinery. Although further *in vitro* studies using all purified components are necessary to test our hypothesis and underlying molecular mechanisms in detail, our results have shed light on the novel aspects of tRNA-mimicking protein complexes.

## SUPPLEMENTARY DATA

Supplementary Data are available at NAR Online.

SUPPORTING INFORMATION
