# Supplementary material for: A genetic approach for analyzing the co-operative function of the tRNA mimicry complex, eRF1/eRF3, in translation termination on the ribosome
Source: Nucleic Acids Res. 2014 Jun 9;42(12):7851–66. doi: 10.1093/nar/gku493 (PMC4081094; doi:10.1093/nar/gku493)
Supplement: SUPPORTING INFORMATION [file supp_42_12_7851__index.html]

A genetic approach for analyzing the co-operative function of the tRNA mimicry complex, eRF1/eRF3, in translation termination on the ribosome — SUPPORTING INFORMATION 

# A genetic approach for analyzing the co-operative function of the tRNA mimicry complex, eRF1/eRF3, in translation termination on the ribosome

## SUPPORTING INFORMATION

**Files in this Data Supplement:**

- Supplemental Figures and Tables
