# Supplementary material for: A genetic approach for analyzing the co-operative function of the tRNA mimicry complex, eRF1/eRF3, in translation termination on the ribosome
Source: Nucleic Acids Res. 2014 Jun 9;42(12):7851–66. doi: 10.1093/nar/gku493 (PMC4081094; doi:10.1093/nar/gku493)
Supplement: SUPPORTING INFORMATION [file supp_gku493_nar-00844-r-2014-File009.pdf]

Supplementary

A Genetic Approach for Cooperative Function of tRNA Mimicry Complex, eRF1/eRF3 in translation termination on the Ribosome

Miki Wada[1,2] and Koichi Ito[2,*]

[1] Technical office, The Institute of Medical Science, The University of Tokyo, Minato-ku, Tokyo, 108-8639, Japan

[2] Department of Medical Genome Sciences, Graduate School of Frontier Sciences, The University of Tokyo, Kashiwa-city, Chiba, 277-8562, Japan

* To whom correspondence should be addressed. Tel: +81 4 7136 3600; Fax: +81 4 7136 3601; Email: itokoichi@k.u-tokyo.ac.jp

SUPPLEMENTARY TABLE AND FIGURES LEGENDS

Table S1. List of *S. cerevisiae* strains used in this study.

Table S2. List of vectors and plasmids used in this study.

Table S3. List of primers used in this study.

Figure S1

A) Schematic drawing of domain structures and conserved regions between Pc-eRF3 and aEF1α. The conserved G-domain motifs are shown as black squares according to ref. 11.

B) Sequence alignment of Pc-eRF3 and its homologs. Muscle (67, 68) and ESPript (http://espript.ibcp.fr/ESPript/ESPript/index.php) (69) programs were used to generate the alignment with secondary structure of aEF1α. Sc-eRF3: *Saccharomyces cerevisiae* eRF3, Sp-eRF3: *Schizosaccharomyces pombe* eRF3, Hs-eRF3: human GSPT1, Ap-EF1a: *Aeropyrum pernix* EF1α, Ec-EFTu: *E. coli* EF-Tu, Ta-EFTu: *Thermus aquaticus* EF-Tu. The Pc-eRF3 mutation sites identified in this study are indicated by triangles.

Figure S2

Sequence alignment of Sc-eRF1 and its homologs. Muscle (67, 68) and ESPript (http://espript.ibcp.fr/ESPript/ESPript/index.php) (69) programs were used to generate the alignment and secondary structure prediction. Sp: *Schizosaccharomyces pombe*, Hs: human, aRF1: *Aeropyrum pernix* aRF1. The Sc-eRF1 cooperative mutation sites identified in this study are indicated by triangles.

Figure S3

Complementation analysis of eRF3 temperature sensitive strain (eRF3ts) of *S. cerevisiae* (YK21-02). Growth of the eRF3ts strain that had been transformed with the Pc-eRF3-mutant plasmids at restrictive temperature, 37℃, is shown. (-: no growth, +: weak growth, ++: good growth)

Figure S4

Yeast two-hybrid binding assay of Pc-eRF3c mutants against Sc-eRF1. Binding domain vector pGBT9 alone, or fused to Sc-eRF3c, Pc-eRF3c^{wt}, or its mutant genes were introduced into the AH109 strain together with pGAD424-Sc-eRF1, and growth of the transformants were examined on selection plates. (-: no binding, +: weak binding, ++: good binding) This figure is complete

data set of Figure 2B.

Figure S5

Complementation analysis of the double tet-OFF strain of *S. cerevisiae* (Y138) by Pc-eRF3c mutants. p416GPD vector, p416GPD-Sc-eRF3c, p416GPD-Pc-eRF3 c^{wt} and its mutants were introduced into Y138 in combination with p414GPD-Sc-eRF1, and growth of the transformants were examined in the presence of 7.5 µg/ml doxycycline. (-: no growth, +: weak growth, ++: good growth)

Figure S6

Yeast two-hybrid binding assay of Sc-eRF1 mutants against Pc-eRF3c^{wt}. Activation domain vector pGAD424 alone, or fused to Sc-eRF1 or its mutant genes were introduced into the AH109 strain together with pGBT9-Pc-eRF3c^{wt}, and growth of the transformants were examined on selection plates. (-: no binding, +: weak binding, ++: good binding) . This figure is complete data set of Figure 4C.

SUPPLEMENTARY REFERENCES

67. Edgar, R.C. (2004) MUSCLE: multiple sequence alignment with high accuracy and high throughput. *Nucleic Acids Res.* **32**(5):1792-1797.

68. Edgar, R.C. (2004) MUSCLE: a multiple sequence alignment method with reduced time and space complexity. *BMC Bioinformatics*, (**5**) 113.

69. Gouet, P., Robert, X. and Courcelle E. (2003) "ESPript/ENDscript: extracting and rendering sequence and 3D information from atomic structures of proteins". Nucl. Acids Res. 31(13), 3320-3323

70. Kikuchi, Y., Shimatake, H. and Kikuchi, A. (1988) A yeast gene required for the G1-to-S transition encodes a protein containing an A-kinase target site and GTPase domain. Embo J, 7, 1175-1182.

71. Mumberg, D., Muller, R. and Funk, M. (1995) Yeast vectors for the controlled expression of heterologous proteins in different genetic backgrounds. Gene, 156, 119-122.

72. Fields, S. and Song, O. (1989) A novel genetic system to detect protein–protein interaction. Nature 340, 245–246.

Table S1. Yeast strains

Y40	MATα ade2D::hisG his3D200 leu2D0 lys2D0 met15D0 trp1D63 ura3D0 *SUP35::HIS3* HO::tTA-tetO (YDR172W/SUP35):: kanMX4	This work
YK21-02	MATα *gst1-1*(*sup35ts*)	(70)
Y138	MATα ade2D::hisG his3D200 leu2D0 lys2D0 met15D0 trp1D63 ura3D0 *SUP35::HIS3* HO::tTA-tetO (YDR172W/SUP35):: kanMX4 tTA-tetO (YBR143C/SUP45)::hphMX4	This work
Y133	MATα *can1-100 ade2-101* his3Δ200 leu2Δ0 lys2Δ0 met15Δ0 trp1Δ63 ura3Δ0 sal2(sup35ts) HO::Rluc-[UGA]-Luc2	This work
Y134	MATα can1-100 ade2-101 his3Δ200 leu2Δ0 lys2Δ0 met15Δ0 trp1Δ63 ura3Δ0 sal2(sup35ts) HO::Rluc-[UGG]-Luc2	This work
S13-I01	MATα ura3-1 ade2-1 leu2-3,112 sal4-2 (sup45ts) HO::Rluc-[UAA]-Luc2	(47)
S13-I03	MATα ura3-1 ade2-1 leu2-3,112 sal4-2 (sup45ts) HO::Rluc-[UAG]-Luc2	(47)
S13-I05	MATα ura3-1 ade2-1 leu2-3,112 sal4-2 (sup45ts) HO::Rluc-[UGA]-Luc2	(47)
S13-I07	MATα ura3-1 ade2-1 leu2-3,112 sal4-2 (sup45ts) HO::Rluc-[UGG]-Luc2	(47)
AH109	MATa *trp1-901 leu2-3 112 ura3-52 his3-200 gal4Δ gal80Δ* *LYS2*::GAL1$_{UAS}$-GAL1$_{TATA}$-HIS3 GAL2$_{UAS}$-GAL2$_{TATA}$-ADE2 *URA3*::MEL1$_{UAS}$-MEL1$_{TATA}$-lacZ, MEL1	CLONTECH

Table S2. Vectors and plasmids

S. cerevisiae expression vectors		
P416CYC	CYC promoter CEN6/ARSH4 URA3	(71)
P416GPD	GPD promoter CEN6/ARSH4 URA3	(71)
P414CYC	CYC promoter CEN6/ARSH4 TRP1	(71)
P414GPD	GPD promoter CEN6/ARSH4 TRP1	(71)
p416GPD-FLAG	p416GPD vector containing FLAG-tag gene for N-terminal fusion	This study
p416CYC-FLAG	p416CYC vector containing FLAG-tag gene for N-terminal fusion	This study
pGAD424	Two-hybrid DNA activation domain vector, GAL4AD (aa768-881)	(72) CLONTECH
pGBT9	Two-hybrid DNA binding domain vector, GAL4BD (aa1-147)	(72) CLONTECH

plasmids	
p416GPD-full-length Pc-eRF3	Pc-eRF3 coding gene (nt 1-1890) in p416GPD
p416GPD-Pc-eRF3c	Pc-eRF3 coding gene (nt 592-1890) with ATG codon in p416GPD
p416CYC-Sc-eRF3c	Sc-eRF3 coding gene (nt 760-2058) in p416CYC
p416GPD-Sc-eRF3c	Sc-eRF3 coding gene (nt 760-2058) in p416GPD
p416GPD-FLAG-full-length Pc-eRF3	Pc-eRF3 coding gene (nt 1-1890) in p416GPD-FLAG
p416GPD-FLAG-Pc-eRF3c	Pc-eRF3c coding gene (nt 592-1890) in p416GPD-FLAG
p416CYC-FLAG-Sc-eRF3c	Sc-eRF3c coding gene (nt 592-1890) in p416CYC-FLAG
p414GPD-Sc-eRF1	Sc-eRF1 in p414GPD
p416CYC-Sc-eRF1	Sc-eRF1 in p416CYC
pGBT9-Pc-eRF3c	Pc-eRF3c coding gene (nt 592-1890) in pGBT9
pGBT9-Pc-eRF3△1	Pc-eRF3 coding gene (nt 1294-1890) in pGBT9
pGBT9-Pc-eRF3c K349A	Pc-eRF3 coding gene (nt 592-1890) K349A in pGBT9
pGBT9-Sc-eRF3c	Sc-eRF3 coding gene (nt 760-2058) in pGBT9
pGAD424-Sc-eRF1	Sc-eRF1 in pGAD424

Table S3. Primers

P363	5'-GGGATCCATATGAATTCAGAGGCAAACAACAGTGA-3'
P318	5'-GGGTCGACCTACTCCTCGGAGATAAGTT-3'
P362	5'-GGGATCCATATGTATGGAAAAGAACACGTAAATG-3'
P555	5'-GGGATCCATATGCCATTAATCATTCCTATTCA-3'
P549	5'-ATTGTAGCAATTAATGCAATGGATGATCCTAC-3'
P550	5'-GTAGGATCATCCATTGCATTAATTGCTACAAT-3'
P291	5'-GGGATCCATATGTTTGGTGGTAAAGATCAC-3'
P292	5'-GGCTCGAGTTACTCGGCAATTTTAACAAT-3'
P289	5'-GGGATCCATATGGATAACGAGGTTGAAAAA-3'
P306	5'-GGGTCGACTTAAATGAAATCATAGTCAGATCC-3'
P420	5'-GGTCTAGAACTAGTCCATGGACTACAAAGACGATG-3'
P421	5'-GGGGATCCTGTCGTCATCGTCTTTGTAGTCCATGG-3'

Figure S1

A

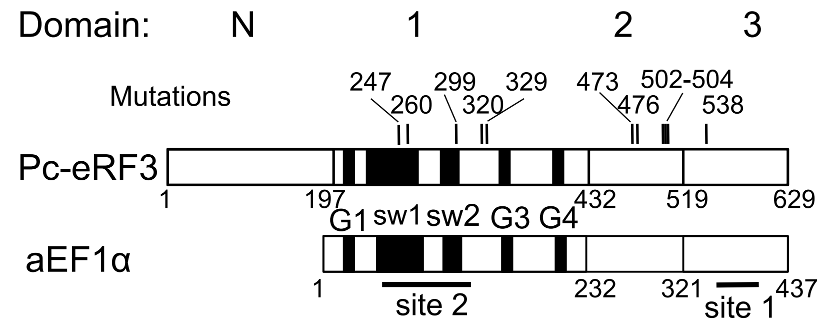
Domain: N 1 2 3
Mutations
247 260 299 320 329 473 476 502-504 538
Pc-eRF3
1 197 432 519 629
G1 sw1 sw2 G3 G4
aEF1α
1 232 321 437
site 2
site 1

B

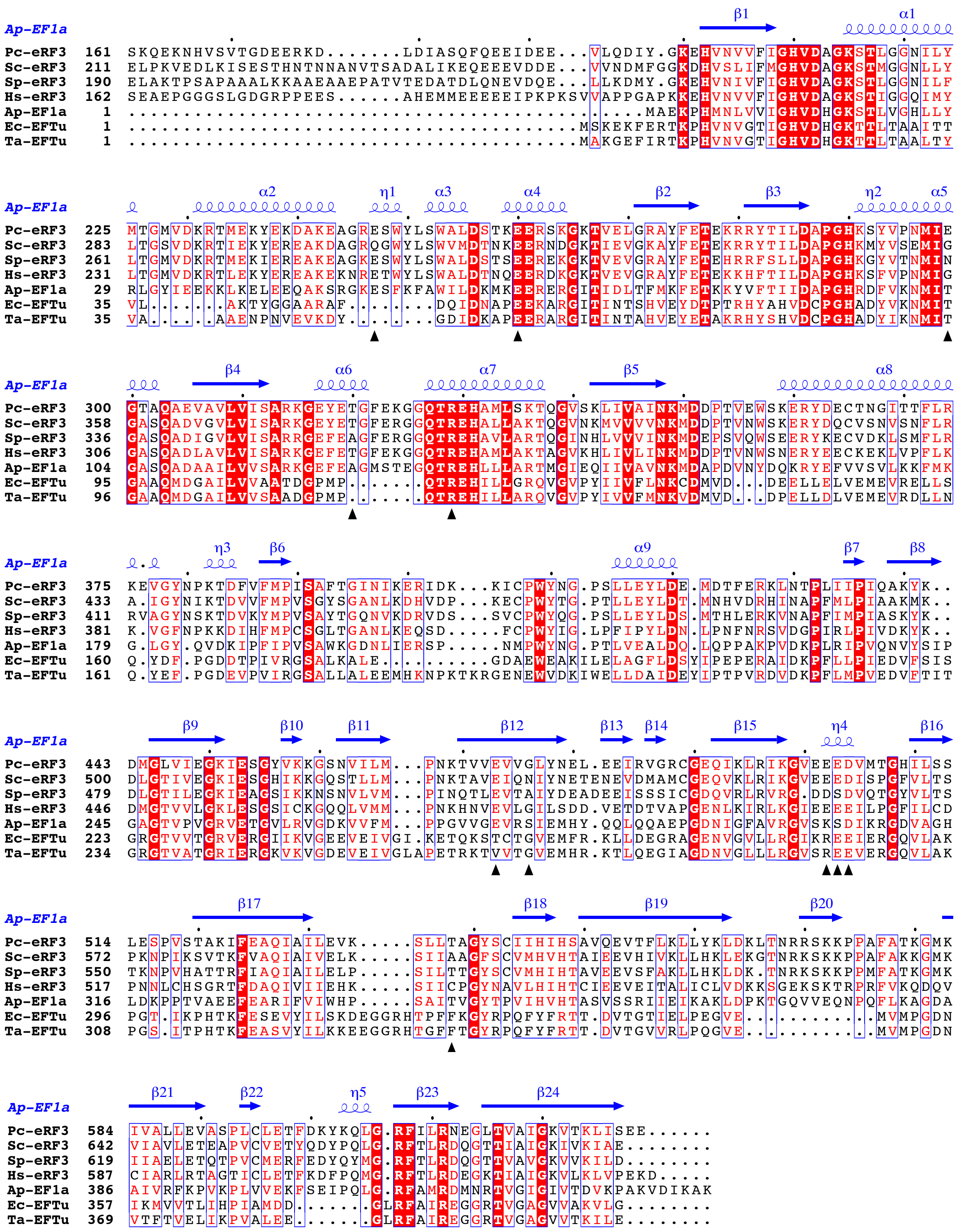
Ap-EF1a β1 α1
Pc-eRF3 161 SKQEKNHVSVTGDEERKD.......LDIASQFQEEIDEE...VLQDIY.GKEHVNVVFIGHVDAGKSTLGGNILY
Sc-eRF3 211 ELPKVEDLKISESTHNTNNANVTSADALIKEQEEEVDDE...VVNDMFGGKDHVSLIFMGHVDAGKSTMGGNLLY
Sp-eRF3 190 ELAKTPSAPAAALKKAAEAAEPATVTEDATDLQNEVDQE...LLKDMY.GKEHVNIVFIGHVDAGKSTLGGNILF
Hs-eRF3 162 SEAEPGGGSLGDGRPPEES......AHEMMEEEEEIPKPKSVVAPPGAPKKEHVNVVFIGHVDAGKSTIGGQIMY
Ap-EF1a 1 ....MAEKPHMNLVVIGHVDHGKSTLVGHLLY
Ec-EFTu 1 ....................................MSKEKFERTKPHVNVGTIGHVDHGKTTLTAAITT
Ta-EFTu 1 ....................................MAKGEFIRTKPHVNVGTIGHVDHGKTTLTAALTY
Ap-EF1a α2 η1 α3 α4 β2 β3 η2 α5
Pc-eRF3 225 MTGMVDKRTMEKYEKDAKEAGRESWYLSWALDSTKEERSKGKTVELGRAYFETEKRRYTILDAPGHKSYVPNMIE
Sc-eRF3 283 LTGSVDKRTIEKYEREAKDAGRQGWYLSWVMDTNKEERNDGKTIEVGKAYFETEKRRYTILDAPGHKMYVSEMIG
Sp-eRF3 261 LTGMVDKRTMEKIEREAKEAGKESWYLSWALDSTSEEREKGKTVEVGRAYFETEHRRFSLLDAPGHKGYVTNMIN
Hs-eRF3 231 LTGMVDKRTLEKYEREAKEKNRETWYLSWALDTNQEERDKGKTVEVGRAYFETEKKHFTILDAPGHKSFVPNMIG
Ap-EF1a 29 RLGYIEEKKLKELEEQAKSRGKESFKFAWILDKMKEERERGITIDLTFMKFETKKYVFTIIDAPGHRDFVKNMIT
Ec-EFTu 35 VL......AKTYGGAARAF.........DQIDNAPEEKARGITINTSHVEYDTPTRHYAHVDCPGHADYVKNMIT
Ta-EFTu 35 VA......AAENPNVEVKDY.........GDIDKAPEERARGITINTAHVEYETAKRHYSHVDCPGHADYIKNMIT
Ap-EF1a β4 α6 α7 β5 α8
Pc-eRF3 300 GTAQAEVAVLVISARKGEYETGFEKGGQTREHAMLSKTQGVSKLIVAINKMDDPTVEWSKERYDECTNGITTFLR
Sc-eRF3 358 GASQADVGVLVISARKGEYETGFERGGQTREHALLAKTQGVNKMVVVVNKMDDPTVNWSKERYDQCVSNVSNFLR
Sp-eRF3 336 GASQADIGVLVISARRGEFEAGFERGGQTREHAVLARTQGINHLVVVINKMDEPSVQWSEERYKECVDKLSMFLR
Hs-eRF3 306 GASQADLAVLVISARKGEFETGFEKGGQTREHAMLAKTAGVKHLIVLINKMDDPTVNWSNERYEECKEKLVPFLK
Ap-EF1a 104 GASQADAAILVVSARKGEFEAGMSTEGQTREHLLLARTMGIEQIIVAVNKMDAPDVNYDQKRYEFVVSVLKKFMK
Ec-EFTu 95 GAAQMDGAILVVAATDGPMP.......QTREHILLGRQVGVPYIIVFLNKCDMVD...DEELLELVEMEVRELLS
Ta-EFTu 96 GAAQMDGAILVVSAADGPMP.......QTREHILLARQVGVPYIVVFMNKVDMVD...DPELLDLVEMEVRDLLN
Ap-EF1a η3 β6 α9 β7 β8
Pc-eRF3 375 KEVGYNPKTDFVFMPISAFTGINIKERIDK...KICPWYNG.PSLLEYLDE.MDTFERKLNTPLIIPIQAKYK..
Sc-eRF3 433 A.IGYNIKTDVVFMPVSGYSGANLKDHVDP...KECPWYTG.PTLLEYLDT.MNHVDRHINAPFMLPIAAKMK..
Sp-eRF3 411 RVAGYNSKTDVKYMPVSAYTGQNVKDRVDS...SVCPWYQG.PSLLEYLDS.MTHLERKVNAPFIMPIASKYK..
Hs-eRF3 381 K.VGFNPKKDIHFMPCSGLTGANLKEQSD.....FCPWYIG.LPFIPYLDN.LPNFNRSVDGPIRLPIVDKYK..
Ap-EF1a 179 G.LGY.QVDKIPFIPVSAWKGDNLIERSP.....NMPWYNG.PTLVEALDQ.LQPPAKPVDKPLRIPVQNVYSIP
Ec-EFTu 160 Q.YDF.PGDDTPIVRGSALKALE........GDAEWEAKILELAGFLDSYIPEPERAIDKPFLLPIEDVFSIS
Ta-EFTu 161 Q.YEF.PGDEVPVIRGSALLALEEMHKNPKTKRGENEWVDKIWELLDAIDEYIPTPVRDVDKPFLMPVEDVFTIT
Ap-EF1a β9 β10 β11 β12 β13 β14 β15 η4 β16
Pc-eRF3 443 DMGLVIEGKIESGYVKKGSNVILM...PNKTVVEVVGLYNEL.EEIRVGRCGEQIKLRIKGVEEEDVMTGHILSS
Sc-eRF3 500 DLGTIVEGKIESGHIKKGQSTLLM...PNKTAVEIQNIYNETENEVDMAMCGEQVKLRIKGVEEEDISPGFVLTS
Sp-eRF3 479 DLGTILEGKIEAGSIKKNSNVLVM...PINQTLEVTAIYDEADEEISSSICGDQVRLRVRG.DDSDVQTGYVLTS
Hs-eRF3 446 DMGTVVLGKLESGSICKGQQLVMM...PNKHNVEVLGILSDD.VETDTVAPGENLKIRLKGIEEEEILPGFILCD
Ap-EF1a 245 GAGTVPVGRVETGVLRVGDKVVFM...PPGVVGEVRSIEMHY.QQLQQAEPGDNIGFAVRGVSKSDIKRGDVAGH
Ec-EFTu 223 GRGTVVTGRVERGIIKVGEEVEIVGI.KETQKSTCTGVEMFR.KLLDEGRAGENVGVLLRGIKREEIERGQVLAK
Ta-EFTu 234 GRGTVATGRIERGKVKVGDEVEIVGLAPETRKTVVTGVEMHR.KTLQEGIAGDNVGLLLRGVSREEVERGQVLAK
Ap-EF1a β17 β18 β19 β20
Pc-eRF3 514 LESPVSTAKIFEAQIAILEVK.....SLLTAGYSCIIHIHSAVQEVTFLKLLYKLDKLTNRRSKKPPAFATKGMK
Sc-eRF3 572 PKNPIKSVTKFVAQIAIVELK.....SIIAAGFSCVMHVHTAIEEVHIVKLLHKLEKGTNRKSKKPPAFAKKGMK
Sp-eRF3 550 TKNPVHATTRFIAQIAILELP.....SILTTGYSCVMHIHTAVEEVSFAKLLHKLDK.TNRKSKKPPMFATKGMK
Hs-eRF3 517 PNNLCHSGRTFDAQIVIIEHK.....SIICPGYNAVLHIHTCIEEVEITALICLVDKKSGEKSKTRPRFVKQDQV
Ap-EF1a 316 LDKPPTVAEEFEARIFVIWHP.....SAITVGYTPVIHVHTASVSSRIIEIKAKLDPKTGQVVEQNPQFLKAGDA
Ec-EFTu 296 PGT.IKPHTKFESEVYILSKDEGGRHTPFFKGYRPQFYFRTT.DVTGTIELPEGVE...........MVMPGDN
Ta-EFTu 308 PGS.ITPHTKFEASVYILKKEEGGRHTGFFTGYRPQFYFRTT.DVTGVVRLPQGVE...........MVMPGDN
Ap-EF1a β21 β22 η5 β23 β24
Pc-eRF3 584 IVALLEVASPLCLETFDKYKQLG.RFILRNEGLTVAIGKVTKLISEE......
Sc-eRF3 642 VIAVLETEAPVCVETYQDYPQLG.RFTLRDQGTTIAIGKIVKIAE........
Sp-eRF3 619 IIAELETQTPVCMERFEDYQYMG.RFTLRDQGTTVAVGKVVKILD........
Hs-eRF3 587 CIARLRTAGTICLETFKDFPQMG.RFTLRDEGKTIAIGKVLKLVPEKD.....
Ap-EF1a 386 AIVRFKPVKPLVVEKFSEIPQLG.RFAMRDMNRTVGIGIVTDVKPAKVDIKAK
Ec-EFTu 357 IKMVVTLIHPIAMDD......GLRFAIREGGRTVGAGVVAKVLG........
Ta-EFTu 369 VTFTVELIKPVALEE......GLRFAIREGGRTVGAGVVTKILE........

Figure S2

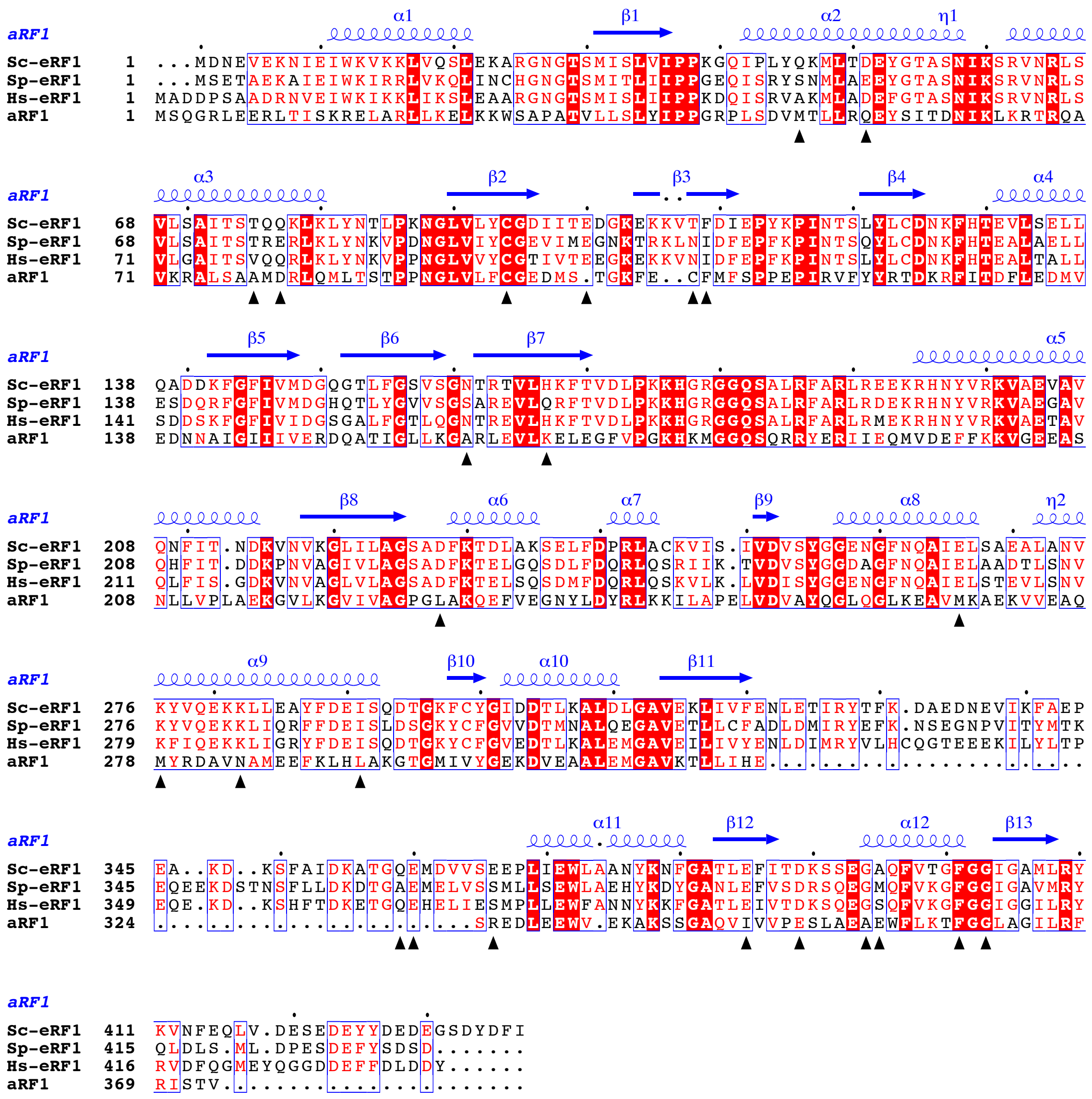

aRF1
α1
β1
α2
η1
Sc-eRF1 1 ...MDNEVEKNIEIWKVKKLVQSLEKARGNGTSMISLVIPPKGQIPLYQKMLTDEYGTASNIKSRVNRLS
Sp-eRF1 1 ...MSETAEKAIEIWKIRRLVKQLINCHGNGTSMITLIIPPGEQISRYSNMLAEEYGTASNIKSRVNRLS
Hs-eRF1 1 MADDPSAADRNVEIWKIKKLIKSLEAARGNGTSMISLIIPPKDQISRVAKMLADEFGTASNIKSRVNRLS
aRF1 1 MSQGRLEERLTISKRELARLLKELKKWSAPATVLLSLYIPPGRPLSDVMTLLRQEYSITDNIKLKRTRQA
aRF1
α3
β2
β3
β4
α4
Sc-eRF1 68 VLSAITSTQQKLKLYNTLPKNGLVLYCGDIITEDGKEKKVTFDIEPYKPINTSLYLCDNKFHTEVLSELL
Sp-eRF1 68 VLSAITSTRERLKLYNKVPDNGLVIYCGEVIMEGNKTRKLNIDFEPFKPINTSQYLCDNKFHTEALAELL
Hs-eRF1 71 VLGAITSVQQRLKLYNKVPPNGLVVYCGTIVTEEGKEKKVNIDFEPFKPINTSLYLCDNKFHTEALTALL
aRF1 71 VKRALSAAMDRLQMLTSTPPNGLVLFCGEDMS.TGKFE..CFMFSPPEPIRVFYYRTDKRFITDFLEDMV
aRF1
β5
β6
β7
α5
Sc-eRF1 138 QADDKFGFIVMDGQGTLFGSVSGNTRTVLHKFTVDLPKKHGRGGQSALRFARLREEKRHNYVRKVAEVAV
Sp-eRF1 138 ESDQRFGFIVMDGHQTLYGVVSGSAREVLQRFTVDLPKKHGRGGQSALRFARLRDEKRHNYVRKVAEGAV
Hs-eRF1 141 SDDSKFGFIVIDGSGALFGTLQGNTREVLHKFTVDLPKKHGRGGQSALRFARLRMEKRHNYVRKVAETAV
aRF1 138 EDNNAIGIIVERDQATIGLLKGARLEVLKELEGFVPGKHKMGGQSQRRYERIIEQMVDEFFKKVGEEAS
aRF1
β8
α6
α7
β9
α8
η2
Sc-eRF1 208 QNFIT.NDKVNVKGLILAGSADFKTDLAKSELFDPRLACKVIS.IVDVSYGGENGFNQAIELSAEALANV
Sp-eRF1 208 QHFIT.DDKPNVAGIVLAGSADFKTELGQSDLFDQRLQSRIIK.TVDVSYGGDAGFNQAIELAADTLSNV
Hs-eRF1 211 QLFIS.GDKVNVAGLVLAGSADFKTELSQSDMFDQRLQSKVLK.LVDISYGGENGFNQAIELSTEVLSNV
aRF1 208 NLLVPLAEKGVLKGVIVAGPGLAKQEFVEGNYLDYRLKKILAPELVDVAYQGLQGLKEAVMKAEKVVEAQ
aRF1
α9
β10
α10
β11
Sc-eRF1 276 KYVQEKKLLEAYFDEISQDTGKFCYGIDDTLKALDLGAVEKLIVFENLETIRYTFK.DAEDNEVIKFAEP
Sp-eRF1 276 KYVQEKKLIQRFFDEISLDSGKYCFGVVDTMNALQEGAVETLLCFADLDMIRYEFK.NSEGNPVITYMTK
Hs-eRF1 279 KFIQEKKLIGRYFDEISQDTGKYCFGVEDTLKALEMGAVEILIVYENLDIMRYVLHCQGTEEEKILYLTP
aRF1 278 MYRDAVNAMEEFKLHLAKGTGMIVYGEKDVEAALEMGAVKTLLIHE........................
aRF1
α11
β12
α12
β13
Sc-eRF1 345 EA..KD..KSFAIDKATGQEMDVVSEEPLIEWLAANYKNFGATLEFITDKSSEGAQFVTGFGGIGAMLRY
Sp-eRF1 345 EQEEKDSTNSFLLDKDTGAEMELVSSMLLSEWLAEHYKDYGANLEFVSDRSQEGMQFVKGFGGIGAVMRY
Hs-eRF1 349 EQE.KD..KSHFTDKETGQEHELIESMPLLEWFANNYKKFGATLEIVTDKSQEGSQFVKGFGGIGGILRY
aRF1 324 ....SREDLEEWV.EKAKSSGAQVIVVPESLAEAEWFLKTFGGLAGILRF
aRF1
Sc-eRF1 411 KVNFEQLV.DESEDEYYDEDEGSDYDFI
Sp-eRF1 415 QLDLS.ML.DPESDEFYSDSD.......
Hs-eRF1 416 RVDFQGMEYQGGDDEFFDLDDY......
aRF1 369 RISTV.......................

Figure S3

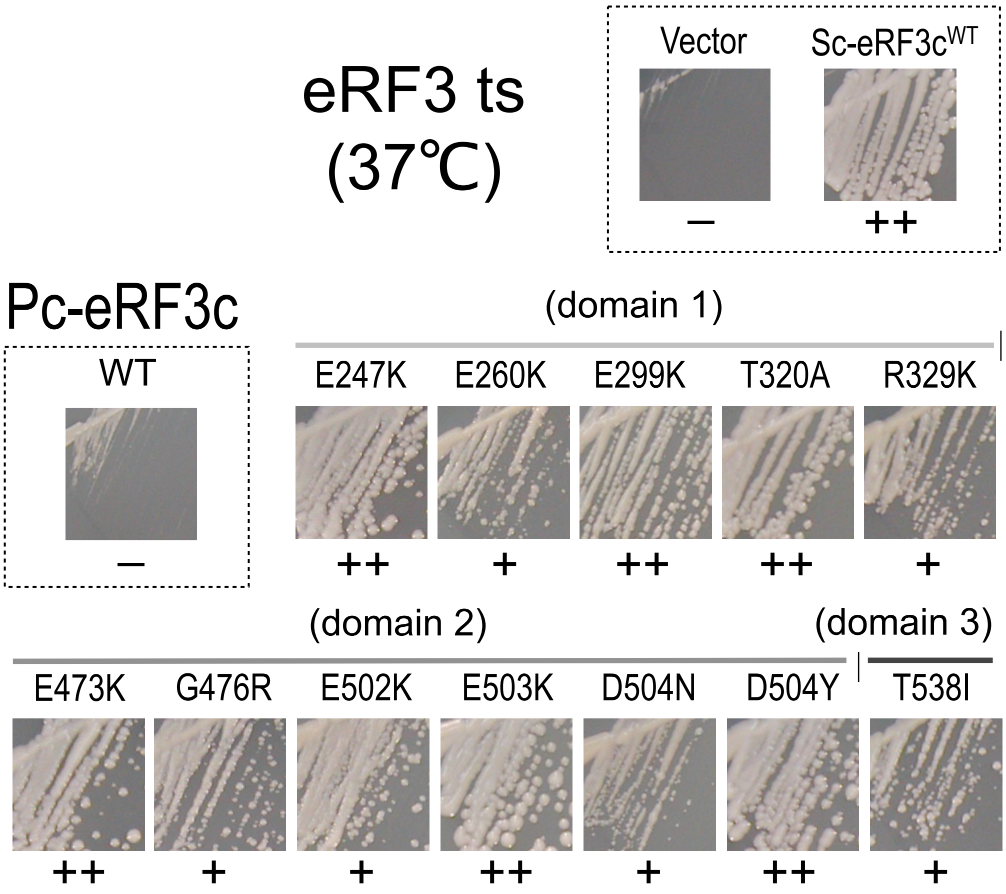
eRF3 ts
(37℃)
Vector
Sc-eRF3cWT
–
++
Pc-eRF3c
WT
–
(domain 1)
E247K
E260K
E299K
T320A
R329K
++
+
++
++
+
(domain 2)
(domain 3)
E473K
G476R
E502K
E503K
D504N
D504Y
T538I
++
+
+
++
+
++
+

Figure S4

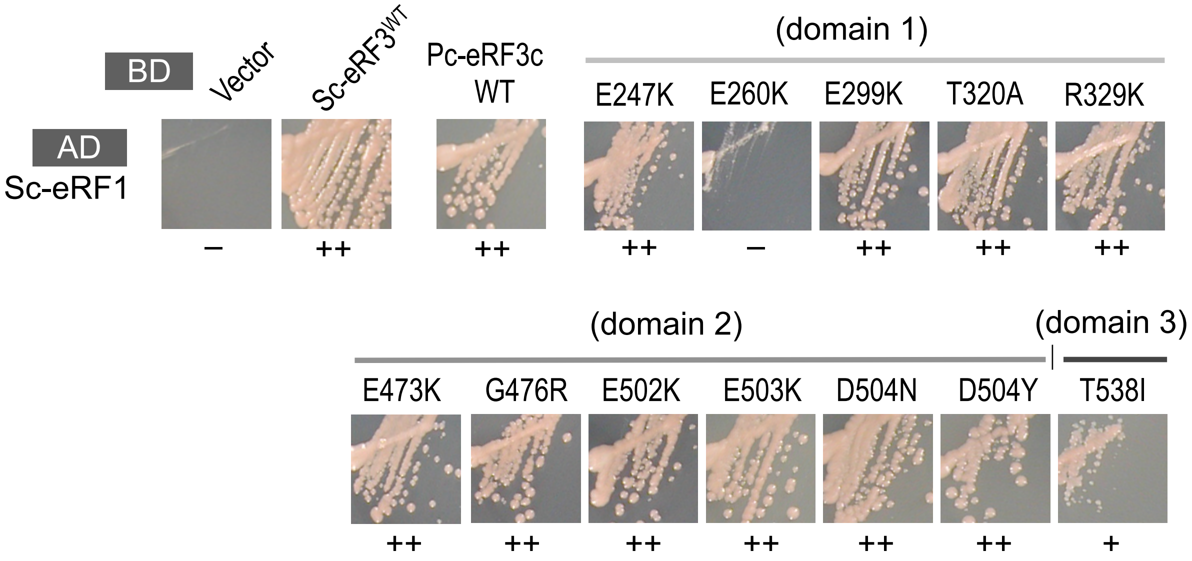
BD
Vector
Sc-eRF3WT
Pc-eRF3c WT
(domain 1)
E247K
E260K
E299K
T320A
R329K
AD
Sc-eRF1
−
++
++
++
−
++
++
++
(domain 2)
(domain 3)
E473K
G476R
E502K
E503K
D504N
D504Y
T538I
++
++
++
++
++
++
+

Figure S5

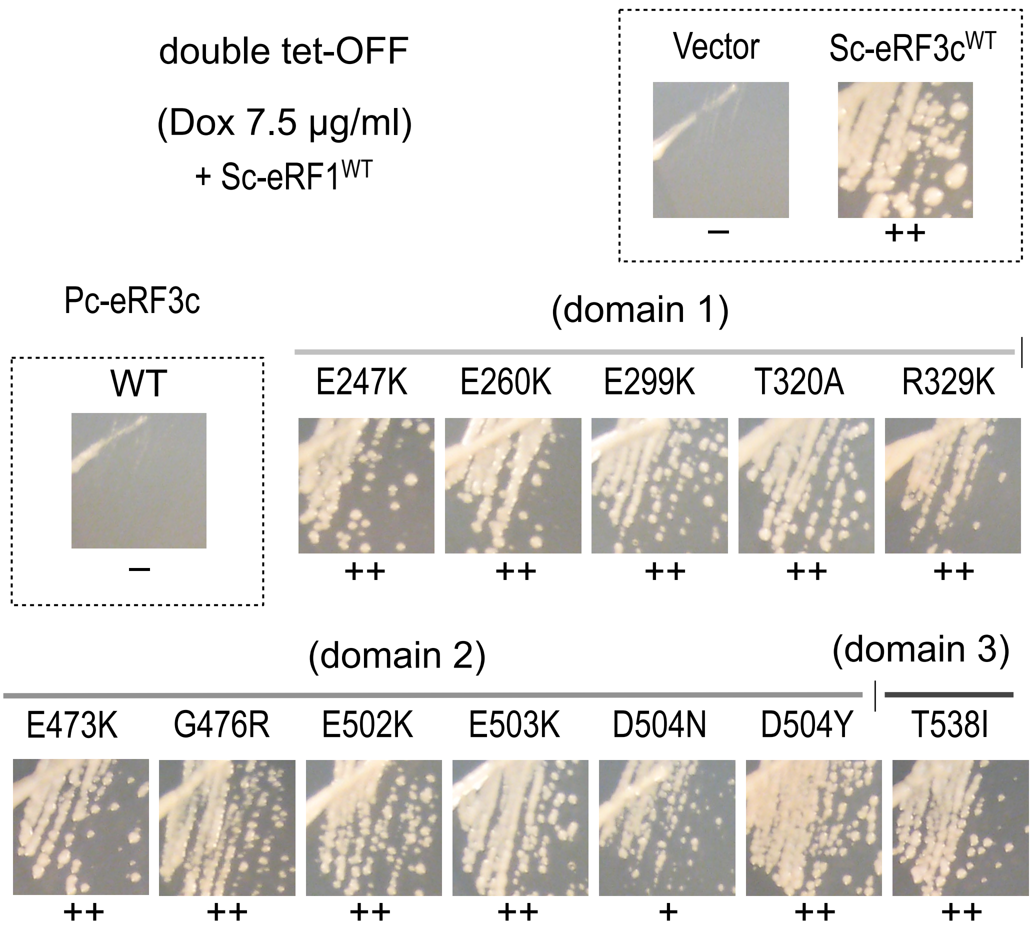

double tet-OFF
(Dox 7.5 μg/ml)
+ Sc-eRF1WT
Vector
Sc-eRF3cWT
–
++
Pc-eRF3c
(domain 1)
WT
–
E247K
E260K
E299K
T320A
R329K
++
++
++
++
++
(domain 2)
(domain 3)
E473K
G476R
E502K
E503K
D504N
D504Y
T538I
++
++
++
++
+
++
++

Figure S6

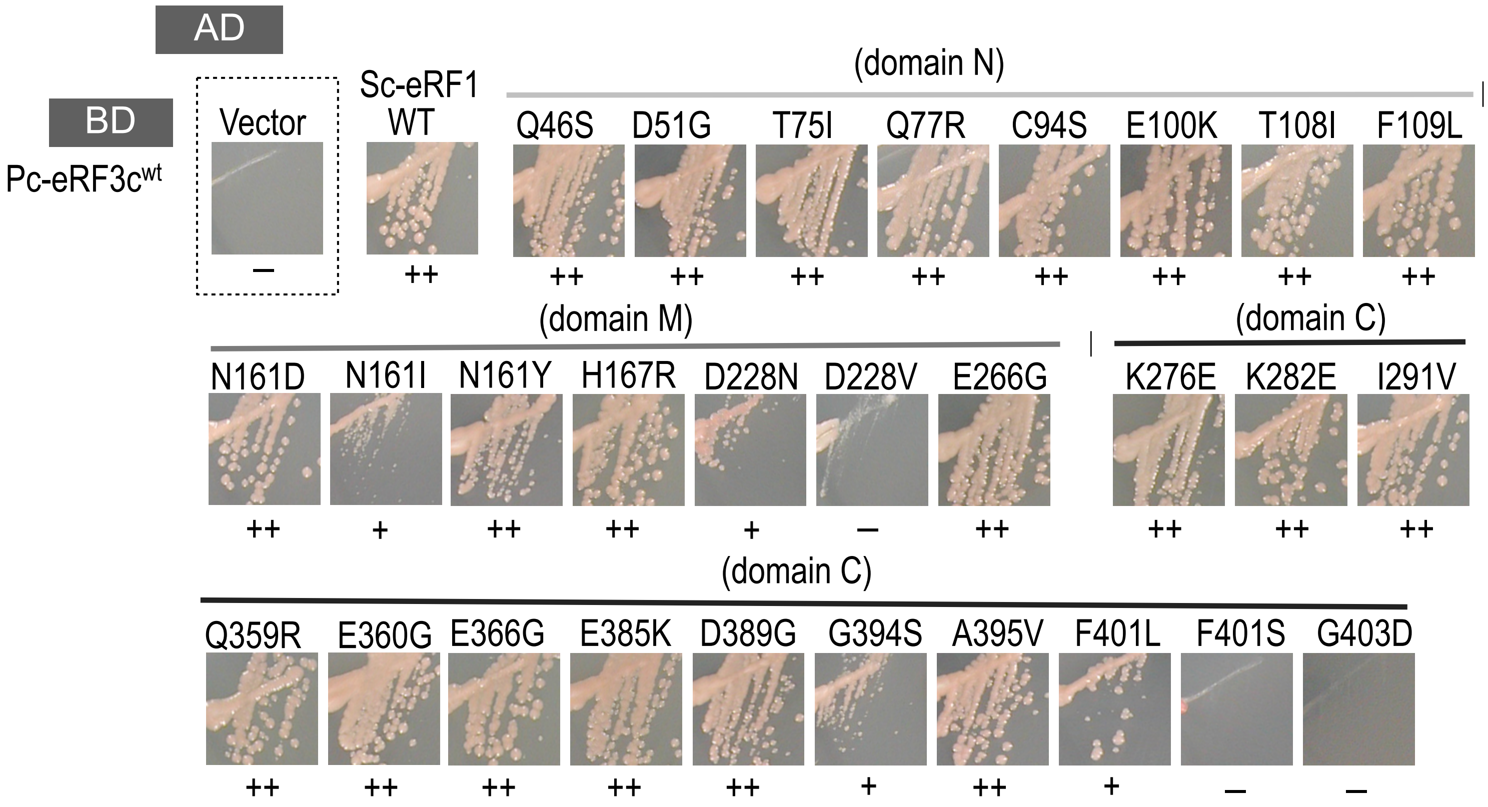
AD
BD
Pc-eRF3c^{wt}
Vector
–
Sc-eRF1
WT
++
(domain N)
Q46S
D51G
T75I
Q77R
C94S
E100K
T108I
F109L
++ ++ ++ ++ ++ ++ ++ ++
(domain M)
N161D
N161I
N161Y
H167R
D228N
D228V
E266G
++ + ++ ++ + – ++
(domain C)
K276E
K282E
I291V
++ ++ ++
(domain C)
Q359R
E360G
E366G
E385K
D389G
G394S
A395V
F401L
F401S
G403D
++ ++ ++ ++ ++ + ++ + – –
